# Novel ICP-OES-Based Method for the Reliable Determination of the Total Content of 15 Elements in Yerba Mate Drinks along with the Determination of Caffeine and the In Vitro Bioaccessibility of the Compounds

**DOI:** 10.3390/molecules28083374

**Published:** 2023-04-11

**Authors:** Maja Welna, Anna Szymczycha-Madeja, Pawel Pohl

**Affiliations:** Division of Analytical Chemistry and Chemical Metallurgy, Faculty of Chemistry, Wroclaw University of Science and Technology, Wybrzeze Wyspianskiego 27, 50-370 Wroclaw, Poland

**Keywords:** yerba mate drinks, mineral content, caffeine, inductively coupled plasma optical emission spectrometry, UV spectrophotometry, multielement analysis, alternative sample preparation procedures, validation, bioaccessibility study, nutritional value

## Abstract

A fully validated inductively coupled plasma optical emission spectrometry (ICP-OES)–based method combined with a simplified sample preparation procedure for the determination of up to 15 elements (Al, Ba, Ca, Cd, Cr, Cu, Fe, K, Mg, Mn, Na, Ni, Pb, Sr, and Zn) in caffeinated yerba mate (YM) drinks was proposed. Various “green” treatments (acidification or dilution with a HNO_3_ solution and direct analysis of untreated YM with or without sonication (US)) that could replace the traditional total sample decomposition before spectrometric measurements were tested and compared. The key selection parameter was the analytical performance of the ICP-OES method obtained with each sample preparation procedure in terms of the precision and the trueness of results and limits of detection (LODs) of elements. It was found that the acidification of YMs with concentrated HNO_3_ to 5%, supported by US (10 min, room temperature (RT)), provided the best results, i.e., LODs at 0.11–8.5 ng g^−1^, precision below 5%, and trueness better than 5% (97.0%–105% as recoveries). Eleven YM drinks, commercially available on the Polish market, were analyzed with the proposed method. In addition to the mineral content, the concentration of caffeine in all analyzed YMs was determined and compared. Finally, the studies were completed by determining the bioaccessible fraction of selected elements and caffeine in YMs using in vitro gastrointestinal digestion (GID) in order to evaluate the nutritional value/risk assessment of these drinks. Accordingly, the bioaccessibility of nutritious elements (Ca, Fe, Mg, Mn, Zn) and caffeine was within 40%–59%. Except for Mn, it was established that by drinking daily 1 L of YMs, the recommended dietary intakes (RDIs) of the aforementioned essential elements were covered to a low degree (<4.5%). Hence, they are not an important source of these elements in the human diet. On the other hand, potentially toxic elements (Al, Ba, Sr) were found in a relatively inert form. Opposite to minerals, YMs can supply human organisms with quite high amounts of natural caffeine in bioaccessible form (31–70 mg *per* serving).

## 1. Introduction

*Ilex paraguariensis* St. Hilaire is a plant belonging to the Aquifoliaceae family that has typically grown and has been cultivated in South America (more specifically in South Brazil, Argentina, Paraguay, and Uruguay) [1,2]. Its dried or roasted green leaves are well known as a source of tealike stimulating beverage with health-promoting properties and energizing effects, commonly named yerba mate (YM) tea or just simply called mate [1,2]. In countries of its origin, mate is consumed in the form of either hot (*chimarrão*) or cold (*tereré*) infusions, both prepared with dried green mate leaves [3]. This cold drink has been appreciated as an excellent low-calorie refreshment and nonalcoholic quenching drink, which is preferable in areas with a hot climate [4]. Finally, mate tea is prepared with roasted leaves and brewed as other herbal teas [3]. Additionally, industrialized drinks and food supplements can also be made from the leaves and stems of this plant [1].

Yerba mate contains numerous biochemically active substances, such as phenolic compounds, methylxanthines (mainly caffeine responsible for stimulating and energizing properties), saponins, flavonoids, vitamins, and essential elements for the human body, including Ca, Mg, K, Fe, and Zn [3,4,5,6]. Therefore, it is not surprising that this plant has been recognized as an important ally in the prevention and development of certain diseases [4,6,7]. Truly, multiple abilities of YM tea such as anti-inflammatory, antioxidant, diuretic, or hepatoprotective, have been proven in in vitro and in vivo studies [4,7].

Today, the popularity of YM has spread wide to other parts of the world, and this tea is marketed, e.g., in Europe, the USA, and Japan, where its consumption is increasing due to awareness about its health properties and beneficial attributes [6,7]. In addition to the traditional drinking of YM in the form of infusions, the extract prepared from the YM infusion began to be an ingredient of alcoholic and nonalcoholic beverages as well as cosmetics, medicines, and food [8,9,10].

Although leafy YM infusions gained popularity in the last few years, nowadays, ready-to-drink beverages of this plant (teas, energy drinks, carbonated soft drinks, and other soft drinks), enhanced by the YM extract, are gathering more and more attention [6,7,9]. Among them, YM-based nonalcoholic beverages meet a consumer’s willingness for functional, healthy, and 100% natural drinks with all “goods” (especially caffeine) sourced from YM leaves. Besides their health and healing properties, such YM drinks could be an excellent alternative to traditional energy drinks supplemented with artificial caffeine.

Regarding substances contained in YM, knowledge about its mineral composition (in terms of total element concentrations) is essential to evaluate the nutritional value and the health risk associated with potentially toxic elements, as well as to assess its quality and safety [4,5,11,12,13]. In this context, since YM is mainly consumed as an infusion, information related to the water solubility of elements from YM leaves and/or commercial dry products should be of particular importance for the exact evaluation of benefits/risks resulting from drinking such beverages [11]. For this purpose, total concentrations of certain elements are usually compared with their nutritional standards, including the recommended daily intake (RDI) and the upper tolerance intake (TUI) for adults, and the intake of these elements *per* serving suggested by the manufacturer is calculated [5,11,13]. However, this may be wrong as the elements are assumed to be 100% available and absorbed by the body during intraoral digestion. In fact, real effects on human health can be assessed only after the estimation of the bioaccessibility of elements by an in vitro digestibility model with artificial enzymes that can be used to mimic physicochemical and enzymatic processes in the human gastrointestinal tract [14,15]. So far, the bioaccessibility of elements from YM is little described in the literature and focuses only on four elements (Al, Cd, Cu, Pb) present in infusions prepared [16]. Therefore, there is still a lack of studies regarding the release of other minerals from YM-based beverages in simulated gastrointestinal conditions to suitably assess their nutritional value.

Despite the high popularity of YM, to our knowledge, no information about the mineral composition of ready-to-drink YM-based beverages has been published in the literature so far. Surprisingly, many studies, although covering a large number of elements (Ag, Al, As, B, Ba, Bi, Be, Ca, Cd, Ce, Co, Cs, Cu, Fe, K, Li, Mg, Mo, Na, Ni, P, Pb, Rb, S, Sb, Se, Sr, Ti, U, V, Zn), are limited to the analysis of commercialized YM [5,10,11,12,16,17,18,19,20,21] and infusions prepared with hot [10,11,12,20,21] or both hot and cold water, simulating this popular beverage [4,5,13,16]. All efforts put into the analysis of YM-based drinks are to assess their physicochemical characteristics [6], antioxidant capacity [6], and the presence of bioactive compounds (phenolic compounds, methylxanthines) [6,9]. The content of elements in YM infusions is measured by applying various spectrometric methods, including flame or graphite furnace atomic absorption spectrometry (F-AAS or GF-AAS) [4,12,21], inductively coupled plasma optical emission spectrometry (ICP-OES) [6,11,14,21], or inductively coupled plasma mass spectrometry (ICP-MS) [10,11], combined with a previous sample treatment, typically based on wet digestion [4,5,13], incineration [20], or acid digestion combined with incineration [5]. Although effective, the abovementioned total digestion procedures are time-consuming, require concentrated reagents, and can lead to losses of analytes and/or contaminations of samples. Hence, works on alternative approaches to the sample preparation prior to spectrometric measurements of elements in YM-based beverages should be of high interest. So far, only a few such studies were carried out in which infusions of YM were directly analyzed [12,21], or a simplified sample preparation, i.e., the dilution of YM infusions with HNO_3_ [10,11] or water [21], was applied.

Therefore, to fill the gap associated with the lack of comprehensive information about the mineral composition of YM-based ready-to-drink beverages, and follow the idea to simplify and/or minimize the steps of the sample preparation of these beverages before their spectrometric measurements, the objective of this work was to develop and validate the green analytical ICP-OES-based method suitable for the sensitive and reliable determination of total concentrations of 15 elements (Al, Ba, Ca, Cd, Cr, Cu, Fe, K, Mg, Mn, Na, Ni, Pb, Sr, and Zn) in caffeinated YM drinks. The suitability of various effortless sample treatments, which, on the one hand, allowed for eliminating the traditional labor-intensive wet digestion, and, on the other hand, ensured the appropriate precision and the trueness of results, and limits of detection (LODs) of elements obtained with ICP-OES, was compared. The usefulness of the proposed methodology was demonstrated in the analysis of 11 YM drinks commercialized in the Polish market. In addition to the mineral composition, the content of caffeine in all analyzed YM drinks was determined by the extraction-spectrophotometric method. Finally, a reliable judgment of the potential harm to the human health associated with the intake of YM drinks was evaluated based on the bioaccessibility of examined compounds after gastrointestinal digestion (GID).

## 2. Results and Discussion

### 2.1. Development of a Simplified Sample Preparation Procedure

We assumed that it is possible to reliably determine 15 elements in YM drinks by ICP-OES with no traditional wet digestion of the samples before their spectrometric measurements. To prove this, several criteria were taken into account: (i) the analytical characteristic of ICP-OES combined with all tested procedures (P1–P7); (ii) the comparison of results (mean concentrations of the studied elements) achieved by ICP-OES for the YM5 drink samples subjected to the alternative sample preparation procedures (P2–P7) with those obtained using the reference sample preparation procedure (P1), i.e., the microwave-assisted closed-vessel digestion in concentrated HNO_3_; and finally (iii), the trueness of results obtained with all sample preparation procedures as assessed by the spike-and-recovery experiments using the standard addition method.

Considering the analytical performance of the ICP-OES method combined with all tested procedures (P1–P7), some validation parameters, i.e., slopes (a), determination coefficients (R^2^) and linearity ranges of calibration curves, limits of determination (LODs) and limits of quantification (LOQs) of elements, and precision (expressed as relative standard deviation, %RSD for replicated (*n* = 3) measurements), were evaluated using 9-point external calibration curves based on simple aqueous (P6, P7) and matrix-matched standard solutions (P1–P5) of the analytes within the concentration range of 0–10.0 mg kg^−1^. Using the slopes obtained for the calibration curves and measuring the standard deviations of procedural blanks (SDs_blank_), the LODs and LOQs (in ng g^−1^) of elements were calculated using the 3 × SD_blank_ and 10 × SD_blank_ criteria, respectively. The masses of the sample portions and the final dilution factors employed in each sample preparation procedure, the respective method LODs (MLODs) and method LOQs (MLOQs), were taken into account.

In the case of the comparison of the results of the multielement analysis of the YM5 drink by ICP-OES obtained using the alternative sample preparation procedures (P2–P7) with those obtained using the reference sample treatment (P1), the average concentrations of the studied elements were taken into account. The significance of differences between the particular results was established using the appropriate statistical tests, i.e., the one-tailed Snedecor–Fisher *F*-test and the two-sample Student’s *t*-test [22]. At first, the differences between the standard deviations (SDs) of these average concentrations were tested at the 95% significance level (α = 0.05) using the *F*-test with the critical value (*F*_critical_) equal to 19.00. This enabled for indicating differences in the precision of the results. When there were no statistically significant differences (*F*_calculated_ < *F*_critical_) between the SDs of the results, the Student’s *t*-test with the critical value (*t*_critical_) of 2.776 (α = 0.05) was used to compare the respective average concentrations of the studied elements. When *F*_calculated_ > *F*_critical_, the Cochran–Cox *C*-test was used with the critical value (*C*_critical_) equal to 4.303 (α = 0.05). Additionally, the precision of the results was expressed also as %RSD.

The use of the spike-and-recovery experiments was intended due to the lack of a suitable liquid CRM having the matrix corresponding to the matrix of the YM drinks. Therefore, this approach was used to verify the trueness of not only the results obtained by ICP-OES combined with the reference sample preparation procedure (P1) but also those obtained when all the alternative sample preparation procedures were used (P2–P7).

#### 2.1.1. Analytical Characteristic of ICP-OES versus Sample Preparation Procedure

The influence of the sample preparation procedure on the analytical characteristics of ICP-OES related to the determination of 15 elements (Al, Ba, Ca, Cd, Cr, Cu, Fe, K, Mg, Mn, Na, Ni, Pb, Sr, Zn) is detailed in Table 1.

In general, regardless of the procedure used, the calibration curves were linear over the entire concentration ranges used for all tested elements. The exception was Sr, for which the linearity occurred only up to a concentration of 4 mg kg^−1^. Additionally, values of the R^2^ coefficient were satisfactory, i.e., ≥0.999. Similarly, differences between the slopes of calibration curves, were rather minor and ranged between 1.0% and 6.7%. Additionally, the analytical signals (three repeated measurements) were acquired with good precision; RSDs from 0.1% to 3.0% were noticed. In the majority of cases, the LODs and LOQs of elements obtained for the sample preparation procedures (P1–P5) were at a similar level (0.10–9.4 ng g^−1^ and 0.33–31 ng g^−1^, respectively); furthermore, these values were comparable for the LODs/LOQs assessed for the direct analysis (P6, P7), i.e., 0.10–10/0.33–33 ng g^−1^. Interestingly, it was observed that among six compared alternative sample treatments (P2–P7), the detectability assessed for the US-assisted procedures (P3, P5, P7) was slightly better (0.10–10 ng g^−1^) than that (0.15–11 ng g^−1^) obtained for the same procedures but without the US treatment (P2, P4, P6). Hence, it seemed that all tested procedures are suitable for the multielement determination of the YM drinks by ICP-OES. However, it failed when referring to the MLODs and MLOQs. In this case, the sample dilution in the particular sample preparation procedures, i.e., 4-fold (P1), 8% (P2, P3), 2-fold (P4, P5), and 0 (P6, P7), was considered. As a result (Table 1), MLODs and MLOQs of elements versus the tested sample preparation procedures differed significantly and changed as follows: P5, P6~P2, P3 < P4, P5 < P1. It was evidenced that a very large sample dilution could be responsible for the nondetection of elements or cause other difficulties with the reliable determination of low concentrations of these elements. In this sense, the sample procedure with the acidification with concentrated HNO_3_ supported by the US treatment (P3) was shown to be the most favorable treatment.

#### 2.1.2. Trueness of Results versus Sample Preparation Procedure

The trueness of the spectrometric measurements of the differently prepared YM5 drink samples was evaluated by comparing the total concentrations of the studied elements obtained using the alternative sample preparation procedures (P2–P7) with those achieved using the reference procedure (P1). The reliability of this procedure (P1) was verified by the analysis of three reference materials (RMs), i.e., two certified reference materials (CRMs): INCT-MPH-2 (mixed Polish herbs) and INCT-TL-1 (tea leaves), and one standard reference material (SRM): SRM 1515 (apple leaves). According to the *t*-test at the 95% level of significance (α = 0.05), it was found that differences between the determined concentrations of elements and certified values were statistically insignificant; i.e., the calculated values of the *t*-test (*t*_calculated_) were lower than the critical value (*t*_critical_) equal to 4.303 [23]. The trueness of results after the application of the reference sample preparation procedure was also verified by the spike-and-recovery experiment using the standard addition method. Consequently, the quantitative recoveries were obtained, i.e., 95.6%–107% (see Table S1), evidencing the absence of losses of the studied elements in this analytical method. This showed the reliability of wet digestion (P1) combined with the ICP-OES analysis of the resulting sample solutions.

The average concentrations of 15 elements determined by ICP-OES in the solutions of the YM5 drink prepared using seven different sample treatments (P1–P7) are listed in Table 2. Simultaneously, in order to statistically compare the results obtained using the reference sample preparation procedure (P1) with those obtained using other alternative sample preparation procedures (P2–P7), the *F*-test and the *t*-test (or the *C*-test) were applied, while the calculated values of these tests, i.e., *F*_calculated_ and ∣*t*_calculated_∣ (or *C*_calculated_), are included in this table.

As shown in Table 2, the tests had to be limited to 9 out of 15 elements studied, i.e., for Al, Ba, Ca, K, Mg, Mn, Na, Sr, and Zn. For the remaining elements, i.e., Cd, Cr, Cu, Ni, and Pb, regardless of the procedure used, the concentrations of these elements were below their MLODs. Additionally, the verification of the validity of the results was impossible in the case of Fe, which could not be determined by ICP-OES combined with the reference sample preparation procedure (P1). It must be commented that it was managed by four alternative sample preparation treatments (P2, P3, P6, P7), i.e., with no (P6, P7) or only with the sample dilution applied in these procedures (P2, P3). Unfortunately, due to a trace concentration of Fe in the YM5 drink, it was not possible to quantify it in more diluted samples obtained with other sample preparation procedures (P1, P4, P5).

Generally, the values of the *F*-test were lower than its critical value (*F*_calculated_ < *F*_critical_). This indicated that the precision of the results achieved using the alternative sample preparation procedures (P2–P7) was the same as that assessed using the reference sample preparation procedure (P1). Therefore, the *t*-test was used to test the significance of differences between the average concentrations of elements determined in the differently prepared samples of the YM5 drink. The only exception was noted for Ca measured in the sample solutions prepared with the preparation procedures P4, P5, and P7. In this case, the calculated values of the *F*-test were higher than the critical value of this test, and hence, the *C*-test was used to compare the respective average concentrations of Ca in these sample solutions.

On the other hand, when considering the precision as %RSD, these values varied on the whole from 0.22% to 1.6% (P1), from 0.88% to 2.9% (P2), from 0.42% to 1.4% (P3), from 0.86% to 4.2% (P4), from 0.65% to 1.6% (P5), from 0.68% to 4.3% (P6), and from 0.53% to 2.8% (P7). Only for Fe and Zn, much higher (>6%) RSDs were obtained, i.e., between 7.1% and 18% (Fe) and 6.2% and 10% (Zn), but the concentration of these elements in the YM5 drink was really low. Nevertheless, the precision of the results of these two elements was the best when the 3-rd procedure was used (P3, acidification with concentrated HNO_3_ supported by the US treatment). As in the case of the LOD and the LOQ of elements, it was observed that the US-assisted sample preparation procedures (P3, P5, P7) provided better precision than that achievable when the US treatment was excluded (P2, P4, P6). Taking into account all the results, the precision of the results obtained for ICP-OES along with the reference sample preparation procedure (P1) and two alternative ones was stated: P3 and P5 were comparable and the best among all the procedures tested.

Considering the average concentrations of the studied elements, the differences between those obtained using the reference sample preparation procedure and the alternative sample preparation procedures were rather small. However, the same relationship was observed, i.e., slightly higher average concentrations of the studied elements when the US-assisted sample preparation procedures (P3, P5, P7) were used. Based on the results of the statistical comparison of the average concentrations of elements determined in the YM5 drink, it was found that only the sample preparation procedure P3 (acidification with concentrated HNO_3_ supported by the US treatment) provided accurate (precise and true) results for all nine elements. In this case, the average concentrations of elements were statistically the same as those obtained using the reference sample preparation procedure (P1). Alternatively, the use of the twofold sample dilution with 10% HNO_3_, followed by the US treatment (P5), seemed to be justified but only in the case of five selected elements (Al, Ba, Mg, Na, Zn); for other elements (Ca, K, Mn, Sr), differences between their average concentrations achieved with both compared sample preparation procedures (P1 and P5) were too high. Unfortunately, the sample preparation procedures P2, P5, P6, and P7 were found to be useless; their use led to incorrect results.

The recoveries of all elements spanned the ranges of 78.0%–106% (P2), 97.0%–105% (P3), 85.5%–115% (P4), 90.9%–110% (P5), 85.1%–114 (P6), and 88.6%–112% (P7) (see Table S1). What is important, independent of the fortification level, is the obtained recovery values corresponded well with the results of the previously performed studies for the alternative sample preparation treatments; i.e., the recoveries obtained for the US-assisted sample preparation procedures (P3, P5, P7) had lower variance values as compared with that established for the remaining sample preparation procedures (P2, P4, P6). Nonetheless, the best results, i.e., the quantitative recoveries of all elements, were achieved using the acidification with concentrated HNO_3_ supported by the US treatment (P3). Satisfactorily, this agreed with the results obtained for the statistical analysis. Hence, this alternative sample preparation procedure (P3) was concluded to successfully replace the traditional wet digestion at the step of the sample preparation of the YM drinks before their multielement analysis by ICP-OES. Consequently, it was used for further studies, i.e., the analysis of the remaining 10 YM drinks.

### 2.2. Application—Multielement Analysis of YM Drinks

The total concentrations of 15 elements determined in the 11 YM drinks by ICP-OES prepared with the aid of their acidification with concentrated HNO_3_ supported next by the US treatment (P3) are presented in Table 3. The results of the caffeine determination are also included in this table. Additionally, the concentration range (min–max) of the determined species along with their averages (as the geometric means) within the examined group of the YM drinks was also included in this table.

The content of Cd and Pb was below their MLODs in all analyzed YM drinks. Similarly, Cr, Cu, and Ni were determined just in 5 (Cr), 6 (Cu), and 9 (Ni) out of 11 YM drinks. For the remaining YM drinks, the concentrations of these elements were lower than the respective MLODs. The precision of measurements was better than 5.0% (0.21%–4.8%). The only exception was found for Cr (YM7, YM8, YM9, YM11), Cu (YM9), Fe (YM5), and Zn (YM1, YM5, YM10), for which RSDs were higher (6.2%–10%). However, this was justified due to the very low concentrations of these elements determined in these samples.

It was established that the concentrations of the studied elements in the analyzed YM drinks were quite differentiated. Differences between the lowest and the highest concentrations varied from 2.5 (Cr) to 88 (K) times. Moreover, significant discrepancies were noticed for Ba, Ni, Sr (1 order of magnitude) and Al, Ca, Fe, K, Mn, and Na (2 orders of magnitude). The highest mineral content (as the sum of the concentrations of all studied elements) was found in the case of the YM6 drink (811 mg kg^−1^ in total), while the lowest was for the YM1 drink and the YM3 drink (44.3 and 57.7 mg kg^−1^, respectively). Hence, to compare the contents of the elements within the whole group of the YM drinks, their geometric means were used. In view of this, Ca, K, Mg, and Na were regarded as the macro elements, while Al, Ba, Cr, Cu, Fe, Mn, Ni, Sr, and Zn were trace elements. In general, the geometric means of the concentrations of the macro elements changed as follows: K > Ca~Mg > Na. The exception was found in the case of YM1, YM4, YM5, and YM10 drinks, for which quite different relations were noticed, i.e., Ca > Na > Mg > K (YM5, YM10), Na > Ca~K~Mg (YM1), and Na > Ca > K > Mg (YM4). The geometric mean of the concertation of K was the highest, i.e., 4 and 7 times higher than that of Ca or Mg and Na, respectively. On the other hand, the geometric means of the concentrations of Ca, Mg, and Na were less discrepant (8.5–23) between the YM drinks as compared with K (88). Considering the trace essential elements, i.e., Cu, Fe, Mn, and Zn, their geometric means of concentrations could be arranged in the following orders: Mn > Fe > Zn > Cu (YM1, YM2, YM4-YM6, YM10) or Mn > Zn > Fe > Cu (YM3, YM7-YM9, YM11). With the exception of Fe, for the other essential trace elements, the differences between the lowest and highest concentrations (6.8–35) were similar to those observed for Ca, Mg, and Na. In the case of Fe, the discrepancy was very high (80) and close to that observed for K. It was also found that the concentrations of Mn were significantly higher (1–2 orders of magnitude) as compared with those determined for the aforementioned essential trace elements. Finally, the geometric means of the concentrations of the rest trace elements (Al, Ba, Cr, Ni, Sr) were low, i.e., 0.194–0.237 mg kg^−1^ (Al, Sr), or very low, i.e., 0.013–0.095 mg kg^−1^ (Ba, Cr, Ni). Moreover, these elements were in the group with quite low variation within the results for all the YM drinks (2.5–18 times).

**Figure 1:**
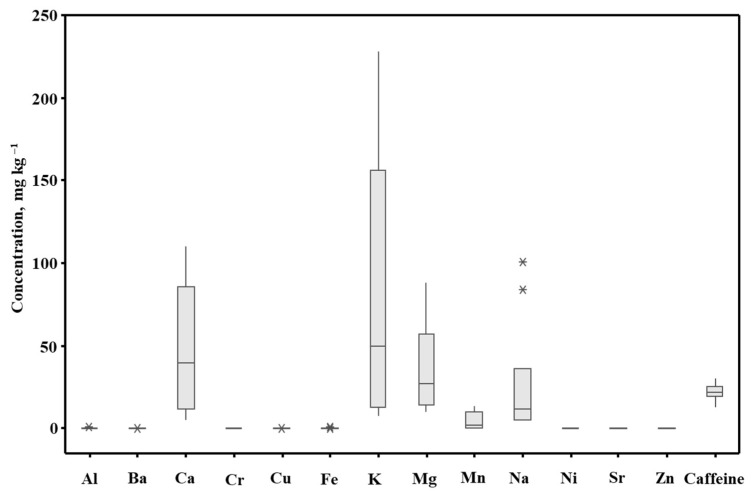
Box-and-whisker plots of the results of the analysis of the yerba mate (YM) drinks. *—an unusually large or small observation.

Furthermore, the data from this table are shown as the box-and-whisker plot (means, medians, minimal and maximal values, in addition to the first and the third quartiles, in mg g^−1^) and illustrated in Figure 1.

Regarding the available literature, the element analysis of yerba mate beverages concerned only the freshly prepared hot and/or cold tea infusions [4,5,10,11,12,13,16,20,21]. Accordingly, this research work reports for the first time the multielement analysis (15) of the commercially available ready-to-drink yerba mate drinks. Despite this, the concentrations of elements determined in all examined YM drinks were within the ranges reported for the fresh YM tea infusions, i.e., Al (0.091–9.00 mg L^−1^ [4,5,11,20,21], Ba (0.139–0.80 mg L^−1^) [5,10,11,20], Ca (27.3–130 mg L^−1^) [5,10,11,12,13,20], Cd (n.d.−0.020 mg L^−1^) [4,5,10,11,12,20], Cu (n.d.−0.90 mg L^−1^) [4,5,10,11,12,20,21], Cr (n.d.−0.050 mg L^−1^) [4,5,10,20,21], Fe (0.055–1.14 mg L^−1^) [4,5,10,11,13,20,21], K (184–1100 mg L^−1^) [5,10,11,12,20], Mn (2.22–18.2 mg L^−1^) [4,5,10,11,20,21], Na (3.87–114 mg L^−1^) [5,12,20], Ni (0.02–0.26 mg L^−1^) [5,10,11,21], Pb (n.d.−0.035 mg L^−1^) [4,5,10,11,20], Sr (n.d.−0.46 mg L^−1^) [5,10,11], Zn (0.08–3.09 mg L^−1^) [4,5,10,11,12,20]. The only exception was noted for Mg, whose concentrations determined in the YM drinks (10.4–88.8 mg kg^−1^) were much lower than those reported by others (73.2–685 mg L^−1^) [5,11,12,13] for the YM tea infusions. Similarly, except for Cu, the values for Al, Cd, and Pb (all determined here and published by the others) were lower than those found by Schmite et al. [16] in the hot and cold infusions of the MY tea, i.e., 11–65 μg g^−1^ (Al), 0.020–0.054 μg g^−1^ (Cd), n.d.−0.29 μg g^−1^ (Pb). The results for Cu (0.040–0.069 μg g^−1^) were congruent.

To confirm the accuracy (precision and trueness) of the spectrophotometric caffeine determination, the recovery test by the standard addition method was carried out (detailed in Section 3.4). As a result, quantitative recoveries were achieved, i.e., 98.1%–101%. Moreover, the precision of measurements was also good, ranging from 0.33% to 2.5% (Table 3). The caffeine content in the YM drinks ranged from 19.3 to 30.3 mg/100 mL (Table 3), excluding the YM7 drink, in which it was almost two times lower than the declared value. Satisfactorily, the results obtained were in very good agreement with the declared values, i.e., 96.7%–113% (as recoveries). For the YM6 and YM8 drinks, the manufacturer declares that these drinks contain “high” (YM6) and “very high” (YM8) amounts of caffeine. Based on the results for all the YM drinks, this could most likely correspond to 20 mg/100 mL (YM6) and 30 mg caffeine/100 mL (YM8). Considering the average concentration of caffeine calculated for all the YM drinks, it matched well the concentration ranges reported for YM beer, soft drinks, novel (functional) YM beverages, and tea infusions [3,6,9].

Additionally, we decided to verify the correctness of the caffeine determination in the case of the YM7 drink due to the discrepancy between the determined and declared caffeine concentrations in this sample. Several additional experiments based on the recovery test were performed for this aim. The caffeine standard was added both before and after the sample extraction. The obtained results (96.1%–99.2%) confirmed that the caffeine content in this drink is likely lower than the declared value. They also proved that caffeine was not lost during the extraction and that there were no matrix effects that could affect the spectrophotometric measurements.

Finally, the relationship between the concentrations of elements and caffeine using Pearson’s correlation coefficient (r) was also investigated. It was carried out according to the study of Kolaylı et al. [23], who found that caffeine binds the metal ions, i.e., Ca, Mg, Fe, Zn, Pb, Mn, Co, and Cr, which suggests its possible chelating activity and ability to form the complex of some certain metals. Unfortunately, no such relations were observed here.

### 2.3. Bioaccessibility of Compounds from YM Drinks

To correctly assess the nutritional value coming from the consumption of the YM drinks, in this work, for the first time, the bioaccessibility study was performed by the in vitro GID procedure (detailed in Section 3.4.2). As far as known, there is a lack of information concerning the release of elements and caffeine from the ready-to-drink YM drinks in the simulated gastrointestinal conditions. Such a study involves only the commercialized YM tea products and their infusions (hot and cold) and focuses only on four elements (Al, Cd, Cu, Pb) [16].

The trueness of the results was verified by carrying out the mass balance. It was based on the comparison of the total content of elements/caffeine (C_t_) determined in the YM drinks with the sum of their concentrations in the dialyzed and nondialyzed fractions, and expressed as the recovery (in %). Taking into account the results of the multielement analysis of the YM drinks (Table 3), the bioaccessibility study was focused on the elements that could be determined in every YM drink, i.e., Al, Ba, Ca, Fe, Mg, Mn, Sr, and Zn. The results of this study are collected in Table 4.

As shown in Table 4, the quantitative recoveries were achieved, i.e., 96.0%–121% and 97.9%–113% for elements and caffeine, respectively. The precision of measurements was also satisfactory; the %RSD values varied from 0.20% to 5.9% (elements) and 0.58% to 6.2% (caffeine). Only for Fe (YM3-YM5) and Zn (YM5, YM10), the precision was worse, i.e., 6.7%–20% due to very low concentrations of these elements (slightly above their MLOQs) in both fractions.

Interestingly, the contributions of the bioaccessible fraction of the individual elements and caffeine in the YM drinks were much less differentiated than the total concentrations of these species; the coefficient of variance (%CV) ranged between 6.2% (caffeine) and 20% (Al). This indicated that the bioaccessibility of the studied elements and caffeine from the YM drinks was quite comparable. Hence, both the arithmetic and geometric means could be used for the comparison of the results within the whole group of YM drinks. However, in this work, consequently, a geometric mean was used. Accordingly, the geometric means of the contributions of the bioaccessible fraction of the examined elements and caffeine varied from 33.3% to 66.0% and 44.6% to 55.2%, respectively. The group of elements with the highest bioaccessibility (with the geometric means of the contributions within 51.9%–59.2%) included Ba, Ca, Sr, and Zn; the contributions of their bioaccessible fraction were between 51.5% and 62.1% (Ba), 45.9% and 60.6% (Ca), 53.8% and 66.0% (Sr), and 42.4% and 59.4% (Zn). The slightly lower bioaccessibility, varied between 42.4% and 54.0% (with a geometric mean of 47.9%) and 39.6% and 49.6% (with a geometric mean of 45.3%), was established for Mg and Mn, respectively. Oppositely, Al and Fe were characterized by the lowest bioaccessibility, i.e., 33.3%–57.1% (Al) and 30.4%–51.4% (Fe). Their geometric mean of the contribution of the bioaccessible fraction was about 40%. The contribution of the bioaccessible fraction of Al was within the range found by Schmite et al. (30%–89%) in the YM tea infusions (hot and cold) [16]. In the case of caffeine, its geometric mean of the contribution of the bioaccessible fraction was close to that for Mg and Mn, i.e., 48.4%; the contribution of the bioaccessible fraction of this compound spanned the range of 44.6%–55.2%.

The results related to bioaccessibility let us estimate the degree of the coverage of the daily requirement for the essential elements involved with the intake of 1 L of the examined YM drinks and thus assess the nutritional value of these drinks. In this case, the concentrations of Ca, Fe, Mg, Mn, and Zn were compared with the dietary reference values (i.e., recommended allowances/adequate daily intakes, RDIs) for healthy adults (male (M) and female (F), aged 31–50), given by the National Research Council [24]. The respective RDIs (in mg day^−1^) were as follows: 1000 (M, F) for Ca, 8 (M) and 18 (F) for Fe, 420 (M) and 320 (F) for Mg, 2.3 (M) and 1.8 (F) for Mn, and 11 (M) and 8 (F) for Zn.

**Figure 2:**
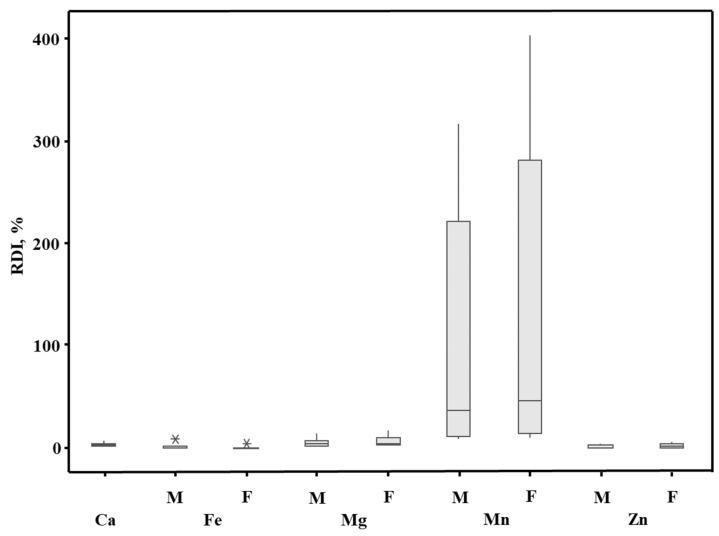
Box-and-whisker plots of the nutritional value (as %RDI) of the analyzed yerba mate (YM) energy drinks (means, medians, minimal and maximal values, in addition to the 1st and the 3rd quartiles). *—an unusually large or small observation.

It was established (Figure 2, Table S2) that 1 L of the YM drinks slightly covered the daily requirements for Ca, Fe, Mg, and Zn, i.e., in 0.20%–4.5% on average. The mean contribution to the realization of their RDI could be arranged as follows: Mg > Ca > Zn > Mn. Accordingly, the RDI standards were covered in 1.7% (M, F) for Ca, 3.5% (M) and 4.5% (F) for Mg, 0.49% (M) and 0.67% (F) for Zn, and 0.44% (M) and 0.20% (F) for Fe. This meant that the examined YM drinks are rather a poor source of the aforementioned elements. However, the exception was the YM6 drink, which is characterized by the highest element contents. The consumption of 1 L of the YM6 drink *per* day could cover the RDI of Ca at 2.4% (M, F), Fe at 7.9% (M) and 3.5% (F), Mg at 13% (M) and 17% (F), and Zn at 4.0% (M) and 5.5% (F). Only Mn appeared to affect the daily realization of its RDI, i.e., at 50% (M) to 64% (F) on average. Although the consumption of 1 L of the YM1–YM5 and YM10 drinks was established to cover the RDI of Mn just at 7.7%–36% (M) and 10%–46% (F), in the case of other YM drinks, the results significantly exceeded the RDI realization for this element. It was 45% (M) and 86% (F) for the YM7 drink, 86% (M) and 138% (F) for the YM9 drink, 121% (M) and 182% (F) for the YM11 drink, 178% (M) and 255% for the YM6 drink, and even 216% (M) and 303% (F) for the YM8 drink. Therefore, the intake of up to 1 L of these YM drinks *per* day could represent a health risk. However, it must be commented that considering their original packaging (330 mL), the realization of the RDI of Mn is reduced to a safe level, i.e., 44%–96% (M) and 56%–122% (F). To conclude, concerning the YM6–YM9 and YM11 drinks, no more than one package should be drunk daily.

Considering the total content of caffeine (Table 3) and assuming the size of the YM drinks, they provide 64–152 mg of caffeine *per* serving. Unfortunately, this calculation may be wrong. In fact, the content of caffeine determined in the bioaccessible fraction is nearly two times lower, i.e., 31–70 mg *per* serving (Table S2). Nevertheless, taking into account the safe limits of caffeine, i.e., 300–400 mg *per* day [6] for healthy adults, it can be said that the examined YM drinks are a good source of natural caffeine; additionally, their intake is within the safe levels for human consumption.

Similarly, the YM drinks did not show any health risks coming from the potentially toxic elements (Al, Ba, Sr) when compared with their tolerance’s upper intake levels (TUI, in mg *per* day), i.e., 65 (Al) [25], 1.3 (Ba) [26], and 9.1 (Sr) [27]. The contributions were between 0.04% and 0.29% (with a geometric mean of 0.15%) for Al, 1.4% and 15% (with a geometric mean of 4.1%) for Ba, and 0.38% and 3.0% (with a geometric mean of 1.3%) for Sr. Accordingly, the intake of these elements via the consumption of even 1 L of the YM drink does not seem to contribute significantly to the respective TUI.

## 3. Materials and Methods

### 3.1. Samples

Ten bottled and one canned lightly carbonated caffeinated YM-based drinks of different brands were purchased from a local market. In total, 11 (*n* = 11) YM drinks were selected and coded as YM1–YM11. Importantly, 9 of them had the caffeine content declared by the producer, which ranges from 20 to 30 mg/100 mL. For the other two, i.e., the YM6 drink and the YM8 drink, only information about “high” (YM6) and “very high” (YM8) caffeine amounts was given. All YM drinks were stored at room temperature in original packings, i.e., 330 mL for the YM1–YM5 drinks or 500 mL for the YM6–YM11 drinks, and were just degassed prior to the sampling in an ultrasonic batch. After opening, they were kept at 4 °C (in a refrigerator); however, due to the limited stability of all YM drinks after opening (up to 48 h), all sample preparations were made within 2 days.

### 3.2. Reagents and Solutions

All reagents used were of analytical-grade purity. A 65% (*m*/*v*) HNO_3_ solution from Merck (Merck, Darmstadt, KGaA, Germany) was used for the sample preparation, i.e., digestion, acidification, and dilution with low concentrated HNO_3_. A Merck Certipur^®^ (Merck) (Burlington, MA, USA) multielemental stock (1000 mg L^−1^) ICP standard solution No. IV was used to prepare the working standard solutions (simple aqueous and matrix-matched regarding the HNO_3_ content) to calibrate an ICP-OES instrument. A caffeine powder (98.5%), ReagentPlus (Acros Organics^TM^, Bridgewater, NJ, USA); a 1 mol L^−1^ NaOH solution (prepared from NaOH pellets (Sigma-Aldrich, St. Louis, MO, USA)); and CH_2_Cl_2_ (Merck) were used for the caffeine determination, and applied for preparing the working standard solutions of caffeine, alkalizing the samples, and extracting caffeine from these samples, respectively. Freshly prepared solutions of the simulated gastric (SGJ) and intestinal (SIJ) juices were used for in vitro gastrointestinal digestion (GID) aiming at determining the purpose of bioaccessible fraction of the studied elements and caffeine. Accordingly, these juices contained 0.32% (*m*/*v*) pepsin with 0.20% (*m*/*v*) NaCl in 0.08 mol L^−1^ HCl (SGJ) and 0.40% (*m*/*v*) pancreatin with 2.5% (*m*/*v*) bile salts in 0.10 mol L^−1^ NaHCO_3_ (SIJ). All solid reagents essential for GID, i.e., pepsin from porcine gastric mucosa (800–2500 units/mg of protein), pancreatin from porcine pancrease, bile salts, PIPES (piperazine-NN-bis(2-ethane-sulfonic acid) disodium salt), NaCl, and NaHCO_3_, as well as 37% (*m*/*v*) HCl, were purchased from Merck. To separate the bioaccessible fraction of the studied elements from incubates of the analyzed YM drinks, high-retention cellulose dialysis bags of 12.4 kDa MWCO (Sigma-Aldrich) were used. Deionized water (18.3 MΩ cm^−1^), from a Barnstead^TM^ (Barnstead, NH, USA) EASYpure RF purification system (model D7033), was used throughout.

### 3.3. Instrumentation

Measurements of total concentrations of the studied elements, i.e., Al, Ba, Ca, Cd, Cr, Cu, Fe, K, Mg, Mn, Na, Ni, Pb, Sr, and Zn, were made using an Agilent benchtop optical emission spectrometer (model 720) with an axially viewed Ar-ICP (Agilent Technologies Inc., Santa Clara, CA, USA). It was equipped with a 4-channel peristaltic pump, a high-resolution Echelle-type polychromator with temperature-controlled optics, and a VistaChip II CCD detector. The sample and standard solutions were introduced using a OneNeb^®^ pneumatic concentric nebulizer (Agilent Technologies Inc., Santa Clara, CA, USA) and a single-pass glass cyclonic spray chamber (Agilent). The plasma was sustained in a standard one-piece quartz torch (2.4 mm ID injector tube). Operating instrument settings, recommended by the manufacturer, were applied as follows: 1200 W (RF power); 15.0, 1.5, and 0.75 L min^−1^ (plasma, auxiliary, and nebulizing Ar flow rates, respectively); 15 s (instrument stabilization and sample uptake delays); 10 s (rinse time); 1 s (replicate read time) and 3 (number of replicates); and 0.75 mL min^−1^ (sample flow rate). The following analytical lines were selected: Al (396.1 nm), Ba (455.4 nm), Ca (317.9 nm), Cd (214.4 nm), Cr (267.7 nm), Cu (327.3 nm), Fe (238.2), K (766,4 nm), Mg (285.2 nm), Mn (257.6 nm), Na (589,592), Ni (230,2 nm), Pb (220.3 nm), Sr (407.7 nm), and Zn (213.8 nm). A fitted background mode with 7 points per line profile was applied for the background correction. Nine-point calibration curves were recorded using simple aqueous and matrix-matched standard solutions within the analyte concentration range of 0.10–10.0 mg kg^−1^. The Multiwave PRO microwave reaction system (Anton Paar GmbH, North Ryde, NSW, Austria), equipped with a 24HVT50 rotor with 50 mL PTFE-TFM pressure activated-venting vessels, was used for the closed-vessel microwave-assisted wet digestion of the samples of YM drinks. A JP Selecta (Spain) ultrasonic bath (UltrasonsH) was applied to sonicate the samples of YM drinks at the step of their preparation with tested and compared alternative sample preparation procedures. A temperature-controlled shaking water bath (Elpin, Poland, model 357) was used in the bioaccessibility study for the in vitro GID.

Caffeine was determined by UV spectrophotometry at 276 nm using a Genesys 10 UV–VIS scanning spectrophotometer (Thermo Scientific, Branchburg, NJ, USA) and a quartz cuvette (10.00 mm, Hellma Analytics). The quantification of caffeine was made against a 9-point calibration curve prepared with the standard solutions of the compound (0–20.0 mg L^−1^) and using CH_2_Cl_2_ as a blank.

### 3.4. Sample Preparation Procedures Prior to Analysis

#### 3.4.1. Total Content of Elements

Seven different sample preparation procedures (Ps) prior to the multielement analysis of the YM drinks by ICP-OES were tested. These included the traditional high-temperature closed-vessel microwave (MW)-assisted digestion with concentrated HNO_3_ (P1) as well as nondigestive (alternative) treatments (P2–P7) requiring only minimal manipulations, i.e., the acidification with concentrated HNO_3_ (P2, P3) and the dilution with low concentrated HNO_3_ (P4, P5), as well as no treatment at all (P6, P7). In addition, in the case of P3, P5, and P7, the additional support of the sample preparation, involving the use of the US, was included. The MW-assisted digestion (P1) combined with ICP-OES measurements was taken as the reference method. Among all analyzed YM drinks, the YM5 drink was selected and used in all test studies related to the selection of the adequate sample preparation procedure.

**Microwave-assisted closed-vessel digestion (P1, reference procedure):** Five-gram portions of the YM5 drink were weighed into PTFE vessels and treated with 5.0 mL of concentrated HNO_3_. Samples were digested by employing a 6-step program with a maximum temperature of 190 °C for 60 min. Afterward, the resulting sample digests were quantitatively transferred into 30 mL polypropylene (PP) screw-capped containers (Equimed, Poland) and diluted with deionized water to obtain 20.0 g of sample solutions.

**Simplified nondigestive procedures (P2–P7):** For the acidification (P2, P3), 20.0 g portions of the YM5 drink were weighed into 30 mL PP screw-capped containers and acidified with HNO_3_ (by adding appropriate volumes of the concentrated reagent) to reach its final concentrations of 5% (P2, P3), followed by the additional US treatment in an ultrasonic bath for 10 min at room temperature (RT) in the case of P3. Considering the dilution with low concentrated HNO_3_ (P4, P5), 10.0 g portions of the YM5 drink were weighed into 30 mL PP screw-capped containers and 2-fold (*v/v*) diluted with a 10% (*v/v*) HNO_3_ solution so that its final concentration in the resultant sample solutions was 5%. In the case of P5, the resultant sample solutions were additionally sonicated (10 min, RT). Finally, in the case of the direct analysis (P6, P7), 10.0 g portions of the YM5 drink were placed into 10 mL screw-capped PP tubes and analyzed without any pretreatment. As before (the case of P3 and P5), in the case of P7, one set of untreated samples was also subjected to the US treatment (10 min, RT).

All sample solutions were prepared by weight (to avoid differences in their density) and analyzed in triplicate (*n* = 3). With each set of digested, acidified, and diluted samples, respective procedural blanks were simultaneously run using deionized water instead of the YM5 drink, analyzed, and considered in the final results. These blanks were also used as appropriate diluents for the preparation of the matrix-matched standard solutions for given sample preparation procedures (P1–P5) to match the effect of the HNO_3_ concentration on measurements of the studied elements by ICP-OES. In the case of the direct analysis (P6, P7), deionized water was used as a blank, while the spectrometric measurements were performed against the simple aqueous standards.

Except for Ca, Mg, K, and Na, concentrations of the remaining elements were determined in undiluted sample solutions. To measure the concentrations of the abovementioned elements, the prepared sample solutions were appropriately diluted, i.e., 10- to 100-fold, depending on the procedure used.

#### 3.4.2. Bioaccessible Fraction—In Vitro Gastrointestinal Digestion Procedure

The determination of the bioaccessibility of elements in the YM drinks was made with the use of the in vitro GID. To simulate the in vitro GID, a 2-step procedure with SGJ and SIJ solutions was used [14,15]. In this procedure, 20.0 g portions of each YM drink (*n* = 3) were weighed into 50 mL PP tubs, then adjusted to pH 2.0 (6.0 mol L^−1^ HCl solution was used), and finally filled with 3.0 mL of the SGJ solution to simulate at first the gastric digestion. Next, the samples were incubated in a shaking water bath at 37 °C with agitation (150 rpm) for 2 h. After this time, the tubes were placed into an ice bath (10 min) to stop the enzymatic reaction. Second, 5.0 mL of the SIJ solution was added to the tubes to simulate the intestinal digestion. Simultaneously, the dialysis membrane bags, filled with 20 mL of a 0.15 mol L^−1^ PIPES solution (pH 7.5 adjusted with HCl), were placed inside these tubes, and the incubation was continued for the next 2 h. As before, the enzymatic reaction was stopped in the ice bath (10 min). In the end, the contents of the dialysis membrane bags (referring to the dialyzable/bioaccessible fraction, D) and the residual solutions of the tubes (referring to the nondialyzable fraction, ND) were transferred to 30 mL PP containers. In the same way as samples, the respective procedural blanks were prepared (using deionized water instead of the YM5 drink). The obtained sample solutions were subjected to multielement analysis by ICP-OES against the external calibration with matrix-matched standard solutions (0–10.0 mg kg^−1^). In addition, UV spectrophotometry was used to determine caffeine in the prepared sample solutions against simple standard solutions of caffeine in CH_2_Cl_2_ (up to 20 mg L^−1^) after the extraction of the target compound into CH_2_Cl_2_. Percentage contributions of the bioaccessible fraction of both the elements and caffeine (in %) contained in the YM drinks were calculated using the following formula: (D/C_t_) × 100, where D corresponded to the concentration of an element/caffeine determined in the dialyzable fraction of the YM drinks and C_t—_to its total concentration determined in these drinks.

#### 3.4.3. Caffeine Content—Solvent Extraction and Detection

The determination of caffeine in all the YM drinks was preceded by the separation of the compound from the alkalized samples using the liquid–liquid solvent extraction with CH_2_Cl_2_ as an extractant. Taking into account the caffeine content in the YM drinks (20–30 mg/100 mL), all of them were diluted (5-fold) before sampling. Next, 5.00 mL of diluted samples (*n* = 3) and water (treated as blank) were alkalized with 1 mol L^−1^ NaOH to pH > 12, transferred to a separatory funnel, and extracted with CH_2_Cl_2_ by shaking for 1 min with the 1st portion (10.0 mL) of the solvent. After the phase separation, the bottom organic phase was transferred into a 25 mL volumetric flask, and the extraction step was repeated 2 times more (10.0 and 5.0 mL of CH_2_Cl_2_). In the end, the flasks with extracted caffeine were diluted with CH_2_Cl_2_ to the mark and measured using a UV–VIS spectrophotometer within the wavelength range of 200–400 nm. The absorption values at 276 nm (wavelength of the maximum caffeine absorption band) and at 320 nm (background) were read. The background-corrected caffeine absorption values were then taken for the final calculations. In the same way as the raw YM drinks, their sample solutions after the GID were analyzed on the caffeine content. However, due to the pH of these solutions, the alkalization step could be omitted.

### 3.5. Spike-and-Recovery Experiments

Due to the lack of a proper certified reference material (CRM), the trueness of the results of the multielement analysis obtained by ICP-OES using the reference sample preparation treatment (P1) as well as all the alternative sample preparation procedures (P2–P7) was checked by spike-and-recovery experiments. Three different concentrations of elements ranging from 0.050 to 0.50 mg kg^−1^ in final sample solutions, added to the YM5 drink samples before their preparation, were tested. The concentration levels of the additions depended on the concentrations of elements determined in the YM5 drink. In the case of Ca, K, Mg, and Na, the additions were made to appropriately diluted YM5 drink samples. Similarly, the results of the determination of caffeine in the YM drink were verified. In this case, samples of the YM5 drink were spiked before the analysis with an aqueous caffeine standard to increase the original concentration of caffeine by 100, 150, and 200 mg L^−1^, and then run through the entire procedure. Recoveries of the added elements and caffeine were calculated by analyzing the unspiked and spiked YM5 drink samples.

## 4. Conclusions

Our work demonstrates for the first time results on the mineral and caffeine content of yerba mate–based drinks determined by ICP-OES and UV–VIS spectrophotometry, respectively. Moreover, we have shown that it is possible to perform multielement analysis with a very simple green sample handling before the spectrometric measurements. The analysis of this kind of beverage after acidification with HNO_3_ to 5%, supported then by the US treatment (10 min, RT), is very simple, reproducible, and dependable, with a precision of 0.21%–4.8% and trueness better than 5% (97.0%–105% as recoveries) and and adequately sensitive (MLODs at 0.11–8.5 ng g^−1^). As shown here, it can be a real alternative to the traditional approach based on wet digestion and successfully used for the routine analysis of the YM drinks at a concentration of up to 15 elements (Al, Ba, Ca, Cd, Cr, Cu, Fe, K, Mg, Mn, Na, Ni, Pb, Sr, Zn) to quickly evaluate their mineral composition. Based on the results, it can be concluded that the 11 YM drinks examined here contain mainly Ca, Mg, K, and Na (16–118 mg kg^−1^ on average) and small amounts of the essential microelements, i.e., Cu, Fe, Mn, and Zn (0.032–2.40 mg kg^−1^ on average), and are free from toxic elements, such as Cd and Pb. They can also be considered a good source of natural caffeine (19.3 to 30.3 mg/100 mL on average). In addition, to reliably judge the potential harm to human health associated with the intake of YM drinks, the bioaccessibility of the examined compounds (both elements and caffeine) after gastrointestinal digestion (GID) was evaluated. Taking into account the bioaccessibility of the nutritious elements from the YM drinks (~50%), it appears that the drinking of 1 L of the YM drinks *per* day rather poorly (<4.5%) covers the respective RDIs of Ca, Fe, Mg, and Zn. The drinking of the YM drinks contributes highly only to the RDI of Mn (~55%). On the other hand, these YM drinks are safe in terms of the presence of potentially toxic elements; the consumption of the YM drinks covers a small percentage (0.15%–4.1%) of Al, Ba, and Sr in their bioaccessibility form. Although the examined YM drinks are rather not an important source of the aforementioned elements in the human diet, they supply human organisms with quite high amounts of natural caffeine. Assuming the bioaccessibility of this compound (~48%) and the original size of the YM drinks (330–500 mL), it was found that they contain 31–70 mg of caffeine *per* serving. Importantly, considering the safe limits of caffeine (300–400 mg *per* day), drinking up to 1 L of the YM drinks does not represent any health problem.

## Figures and Tables

**Table 1:** Selected figures of merit for the multielement analysis of yerba mate (YM) drinks by ICP-OES assessed considering the external calibration with the matrix-matched and simple aqueous standard solutions for all tested sample preparation procedures (P1–P7).

		P1	P2	P3	P4	P5	P6	P7
Al	a ^a^	40,190	40,883	42,130	41,619	40,230	43,033	42,479
	R^2 b^	0.9999	0.9999	0.9999	0.9999	0.9999	0.9995	0.9999
	range ^c^	0–10	0–10	0–10	0–10	0–10	0–10	0–10
	RSD ^d^	0.54	0.67	0.10	0.22	0.92	0.80	0.88
	LOD ^e^	2.0 (8.0) ^g^	1.5 (1.6) ^g^	1.1 (1.2) ^g^	2.6 (5.2) ^g^	2.4 (4.8) ^g^	2.0 (2.0) ^g^	1.5 (1.5) ^g^
	LOQ ^f^	6.7 (27) ^g^	5.0 (5.4) ^g^	3.7 (4.0) ^g^	8.7 (17) ^g^	8.0 (16) ^g^	6.7 (6.7) ^g^	5.0 (5.0) ^g^
Ba	a ^a^	1,471,486	1,417,522	1,450,487	1,335,908	1,276,897	1,445,324	1,306,609
	R^2 b^	0.9997	0.9993	0.9995	0.9992	0.9996	0.9994	0.9994
	range ^c^	0–10	0–10	0–10	0–10	0–10	0–10	0–10
	RSD ^d^	0.21	0.22	1.2	0.39	0.56	1.1	1.4
	LOD ^e^	0.10 (0.40) ^g^	0.13 (0.14) ^g^	0.15 (0.16) ^g^	0.16 (0.32) ^g^	0.15 (0.30) ^g^	0.22 (0.22) ^g^	0.22 (0.22) ^g^
	LOQ ^f^	0.33 (1.3) ^g^	0.43 (0.46) ^g^	0.50 (0.54) ^g^	0.53 (1.1) ^g^	0.50 (1.0)) ^g^	0.73 (0.73) ^g^	0.73 (0.73) ^g^
Ca	a ^a^	36,384	36,027	34,970	35,737	34,645	39,659	38,144
	R^2 b^	0.9999	0.9999	0.9999	0.9999	0.9999	0.9996	0.9995
	range ^c^	0–10	0–10	0–10	0–10	0–10	0–10	0–10
	RSD ^d^	0.26	0.39	0.44	0.15	0.65	0.49	0.72
	LOD ^e^	2.3 (9.2) ^g^	8.0 (8.6) ^g^	6.5 (7.0) ^g^	7.1 (14) ^g^	3.3 (6.6) ^g^	2.3 (2.3) ^g^	2.4 (2.4) ^g^
	LOQ ^f^	7.7 (31) ^g^	27 (29) ^g^	22 (24) ^g^	24 (48) ^g^	11 (22) ^g^	7.7 (7.7) ^g^	8.0 (8.0) ^g^
Cd	a ^a^	36,772	35,485	35,686	36,685	34,301	37,687	37,452
	R^2 b^	0.9999	0.9999	0.9999	0.9998	0.9999	0.9993	0.9998
	range ^c^	0–10	0–10	0–10	0–10	0–10	0–10	0–10
	RSD ^d^	0.37	0.41	0.74	0.10	0.32	0.56	0.55
	LOD ^e^	0.39 (1.6) ^g^	0.48 (0.52) ^g^	0.46 (0.50) ^g^	0.49 (0.98) ^g^	0.43 (0.86) ^g^	0.27 (0.27) ^g^	0.60 (0.60) ^g^
	LOQ ^f^	1.3 (5.2) ^g^	1.6 (1.7) ^g^	1.5 (1.6) ^g^	1.6 (3.2) ^g^	1.4 (2.8) ^g^	0.90 (0.90) ^g^	2.0 (2.0) ^g^
Cr	a ^a^	27,281	25,917	26,199	26,317	25,631	25,895	25,529
	R^2 b^	0.9999	0.9999	0.9999	0.9999	0.9999	0.9996	0.9999
	range ^c^	0–10	0–10	0–10	0–10	0–10	0–10	0–10
	RSD ^d^	0.79	0.24	0.43	0.45	0.38	0.26	0.77
	LOD ^e^	0.84 (3.4) ^g^	0.95 (1.0) ^g^	0.71 (0.80) ^g^	0.73 (1.5) ^g^	0.89 (1.8) ^g^	0.90 (0.90) ^g^	0.68 (0.68) ^g^
	LOQ ^f^	2.8 (11) ^g^	3.2 (3.4) ^g^	2.4 (2.6) ^g^	2.4 (4.8) ^g^	3.0 (6.0) ^g^	3.0 (3.0) ^g^	2.3 (2.3) ^g^
Cu	a ^a^	39,753	38,458	38,405	39,048	38,388	38,091	37,257
	R^2 b^	0.9999	0.9999	0.9999	0.9999	0.9999	0.9997	0.9999
	range ^c^	0–10	0–10	0–10	0–10	0–10	0–10	0–10
	RSD ^d^	0.69	0.55	0.77	0.46	0.54	0.65	1.1
	LOD ^e^	0.54 (2.2) ^g^	1.2 (1.3) ^g^	0.73 (0.80) ^g^	1.4 (2.8) ^g^	0.71 (1.4) ^g^	0.81 (0.81) ^g^	0.78 (0.78) ^g^
	LOQ ^f^	1.8 (7.2) ^g^	4.0 (4.3) ^g^	2.4 (2.6) ^g^	4.7 (9.4) ^g^	2.4 (4.8) ^g^	2.7 (2.7) ^g^	2.6 (2.6) ^g^
Fe	a ^a^	24,710	23,388	23,886	24,615	24,002	23,849	23,326
	R^2 b^	0.9999	0.9999	0.9999	0.9999	0.9999	0.9994	0.9999
	range ^c^	0–10	0–10	0–10	0–10	0–10	0–10	0–10
	RSD ^d^	0.27	0.36	0.43	0.42	0.45	0.81	0.84
	LOD ^e^	0.54 (2.2) ^g^	0.92 (1.0) ^g^	0.62 (0.70) ^g^	0.92 (1.8) ^g^	0.74 (1.5) ^g^	0.88 (0.88) ^g^	0.52 (0.52) ^g^
	LOQ ^f^	1.8 (7.2) ^g^	3.1 (3.3) ^g^	2.1 (2.3) ^g^	3.1 (6.2) ^g^	2.5 (5.0) ^g^	2.9 (2.9) ^g^	1.7 (1.7) ^g^
K	a ^a^	71,023	74,757	76,594	80,209	78,507	85,054	87,271
	R^2 b^	0.9989	0.9993	0.9990	0.9993	0.9993	0.9998	0.9996
	range ^c^	0–10	0–10	0–10	0–10	0–10	0–10	0–10
	RSD ^d^	0.69	0.93	0.79	0.28	1.7	0.63	0.95
	LOD ^e^	2.0 (8.0) ^g^	1.8 (1.9) ^g^	2.3 (2.5) ^g^	1.9 (3.8) ^g^	2.0 (4.0) ^g^	2.9 (2.9) ^g^	1.3 (1.3) ^g^
	LOQ ^f^	6.7 (27) ^g^	6.0 (6.5) ^g^	7.7 (8.3) ^g^	6.3 (13) ^g^	6.7 (13) ^g^	9.7 (9.7) ^g^	4.3 (4.3) ^g^
Mg	a ^a^	38,784	40,565	40,956	42,571	40,759	43,211	42,145
	R^2 b^	0.9999	0.9999	0.9999	0.9999	0.9999	0.9997	0.9999
	range ^c^	0–10	0–10	0–10	0–10	0–10	0–10	0–10
	RSD ^d^	0.33	0.58	0.70	0.68	0.42	0.82	1.1
	LOD ^e^	0.92 (3.7) ^g^	2.7 (2.9) ^g^	2.3 (2.5) ^g^	0.93 (1.9) ^g^	0.87 (1.7) ^g^	2.7 (2.7) ^g^	1.8 (1.8) ^g^
	LOQ ^f^	3.1 (12) ^g^	9.0 (9.7) ^g^	7.7 (8.3) ^g^	3.1 (6.2) ^g^	2.9 (5.8) ^g^	9.0 (9.0) ^g^	6.0 (6.0) ^g^
Mn	a ^a^	262,146	256,897	260,614	260,614	215,639	253,719	258,973
	R^2 b^	0.9999	0.9998	0.9999	0.9997	0.9999	0.9994	0.9999
	range ^c^	0–10	0–10	0–10	0–10	0–10	0–10	0–10
	RSD ^d^	1.6	2.2	1.7	0.94	2.6	0.91	2.8
	LOD ^e^	0.10 (0.40) ^g^	0.33 (0.36) ^g^	0.19 (0.21) ^g^	0.15 (0.30) ^g^	0.10 (0.20) ^g^	0.35 (0.35) ^g^	0.14 (0.14) ^g^
	LOQ ^f^	0.33 (1.3) ^g^	1.1 (1.2) ^g^	0.63 (0.68) ^g^	0.50 (1.0) ^g^	0.33 (0.66) ^g^	1.2 (1.2) ^g^	0.47 (0.47) ^g^
Na	a ^a^	196,714	217,996	210,142	222,108	194,598	218,742	217,587
	R^2 b^	0.9990	0.9996	0.9994	0.9997	0.9998	0.9999	0.9999
	range ^c^	0–10	0–10	0–10	0–10	0–10	0–10	0–10
	RSD ^d^	0.29	0.58	0.50	0.19	1.2	0.91	1.5
	LOD ^e^	2.5 (10) ^g^	4.9 (5.3) ^g^	2.5 (2.7) ^g^	0.66 (1.3) ^g^	1.3 (2.6) ^g^	4.3 (4.3) ^g^	0.87 (0.87) ^g^
	LOQ ^f^	8.3 (33) ^g^	16 (17) ^g^	8.3 (9.0) ^g^	2.2 (4.4) ^g^	4.3 (8.6) ^g^	14 (14) ^g^	2.9 (2.9) ^g^
Ni	a ^a^	6636	6143	5924	6077	5983	6249	6350
	R^2 b^	0.9999	0.9998	0.9999	0.9998	0.9999	0.9996	0.9998
	range ^c^	0–10	0–10	0–10	0–10	0–10	0–10	0–10
	RSD ^d^	0.57	0.26	0.58	0.28	0.84	1.3	0.87
	LOD ^e^	2.8 (11) ^g^	4.1 (4.4) ^g^	2.9 (3.1) ^g^	3.1 (6.2) ^g^	2.3 (4.6) ^g^	3.4 (3.4) ^g^	3.4 (3.4) ^g^
	LOQ ^f^	9.3 (37) ^g^	14 (15) ^g^	9.7 (10) ^g^	10 (20) ^g^	7.7 (15) ^g^	11 (11) ^g^	11 (11) ^g^
Pb	a ^a^	1854	1823	1817	1816	1823	1865	1837
	R^2 b^	0.9999	0.9999	0.9999	0.9998	0.9999	0.9994	0.9998
	range ^c^	0–10	0–10	0–10	0–10	0–10	0–10	0–10
	RSD ^d^	0.28	1.2	1.2	1.4	1.5	1.2	0.42
	LOD ^e^	8.6 (34) ^g^	9.4 (10) ^g^	7.9 (8.5) ^g^	8.2 (16) ^g^	6.7 (13) ^g^	11 (11) ^g^	10 (10) ^g^
	LOQ ^f^	29 (116) ^g^	31 (33) ^g^	26 (28) ^g^	27 (54) ^g^	22 (44) ^g^	37 (37) ^g^	33 (33) ^g^
Sr	a ^a^	4,158,828	3,956,137	3,817,574	3,877,645	3,704,165	3,776,872	3,751,271
	R^2 b^	0.9995	0.9997	0.9995	0.9997	0.9992	0.9998	0.9997
	range ^c^	0–4	0–4	0–4	0–4	0–4	0–4	0–4
	RSD ^d^	0.31	0.24	0.86	0.29	3.0	1.0	1.7
	LOD ^e^	0.10 (0.40) ^g^	0.11 (0.12) ^g^	0.10 (0.11) ^g^	0.10 (0.20) ^g^	0.10 (0.20) ^g^	0.15 (0.15) ^g^	0.10 (0.10) ^g^
	LOQ ^f^	0.33 (1.3) ^g^	0.37 (0.40)) ^g^	0.33 (0.36) ^g^	0.33 (0.64) ^g^	0.33 (0.64)) ^g^	0.50 (0.50) ^g^	0.33 (0.33) ^g^
Zn	a ^a^	31,116	30,613	30,822	31,726	30,422	35,284	34,036
	R^2 b^	0.9999	0.9999	0.9999	0.9998	0.9999	0.9991	0.9998
	range ^c^	0–10	0–10	0–10	0–10	0–10	0–10	0–10
	RSD ^d^	0.13	0.73	0.41	0.39	0.80	0.34	0.96
	LOD ^e^	0.63 (2.5) ^g^	0.80 (0.86) ^g^	0.43 (0.46) ^g^	0.61 (1.2) ^g^	0.61 (1.2) ^g^	0.82 (0.82) ^g^	0.77 (0.77) ^g^
	LOQ ^f^	2.1 (8.4) ^g^	2.7 (2.9) ^g^	1.4 (1.5) ^g^	2.0 (4.0) ^g^	2.0 (4.0) ^g^	2.7 (2.7) ^g^	2.6 (2.6) ^g^

P1: microwave-assisted closed-vessel digestion in concentrated HNO_3_. P2: acidification with concentrated HNO_3_ to 5%. P3: acidification with concentrated HNO_3_ to 5%, followed by the sonication (US) treatment. P4: 2-fold (1:1. *w/w*) dilution with 10% HNO_3_. P5: 2-fold (1:1. *w/w*) dilution with 10% HNO_3_, followed by the sonication (US) treatment. P6: direct analysis (no pretreatment). P7: direct analysis preceded by sonication (treatment). ^a^ Slope of the calibration curve (in (a.u.)/(mg kg^−1^)). ^b^ Determination coefficient. ^c^ Concentration range of analytes (in mg kg^−1^). ^d^ Relative standard deviation for replicated (*n* = 3) measurements of the analytes’ signal (a 0.50 mg kg^−1^ standard solution was used). ^e^ Limit of detection (in ng g^−1^) in measured sample solutions achievable with ICP-OES combined with different sample preparation procedures (P1–P7). ^f^ Limit of quantification (in ng g^−1^) in measured sample solutions achievable with ICP-OES combined with different sample preparation procedures (P1–P7). ^g^ Method limits of detection (MLOD) and quantification (MLOQ) (in ng g^−1^) for original samples (final dilution factors and sample portions used in respective sample preparation procedures (P1–P6) were included).

**Table 2:** Total concentrations of elements determined in solutions of the YM5 drink using ICP-OES and different sample preparation procedures (P1–P7) along with calculated values of the *F*-test (*F*_calculated_) and the *t*-test (∣*t*_calculated_∣) for the statistical comparison of standard deviations (SDs) of average concentrations of elements and these average concentrations respectively determined using alternative sample treatments (P2–P7) against the reference sample preparation procedure (P1). Significant differences are underlined.

		P1	P2	P3	P4	P5	P6	P7
Al	C_t_ ^a^	0.073 ± 0.001	0.070 ± 0.002	0.073 ± 0.001	0.070 ± 0.002	0.072 ± 0.001	0.069 ± 0.003	0.071 ± 0.002
	*F*_calculated_ ^b^		4.00	1.00	4.00	1.00	9.00	4.00
	∣*t*_calculated_∣ ^c^		2.324	0.000	2.324	1.225	2.191	1.549
Ba	C_t_ ^a^	0.082 ± 0.001	0.075 ± 0.001	0.080 ± 0.001	0.072 ± 0.003	0.079 ± 0.001	0.070 ± 0.003	0.072 ± 0.002
	*F*_calculated_ ^b^		1.00	1.00	9.00	4.00	9.00	4.00
	∣*t*_calculated_∣ ^c^		8.573	2.327	5.477	2.324	6.573	7.746
Ca	C_t_ ^a^	89.7 ± 0.2	88.2 ± 0.8	91.0 ± 0.4	85.1 ± 1.0	88.0 ± 0.7	73.7 ± 1.6	82.1 ± 1.3
	*F*_calculated_ ^b^		16.00	6.25	25.00	12.25	64.00	42.25
	∣*t*_calculated_∣ ^c^		3.151	2.573	6.379 ^d^	4.045	14.033 ^d^	8.172 ^d^
Cd	C_t_^a^	*<1.6* ^e^	*<0.52* ^e^	*<0.50* ^e^	*<0.98* ^e^	*<0.86* ^e^	*<0.27* ^e^	*<0.60* ^e^
Cr	C_t_^a^	*<3.4* ^e^	*<1.0* ^e^	*<0.80* ^e^	*<1.5* ^e^	*<1.8* ^e^	*<0.90* ^e^	*<0.68* ^e^
Cu	C_t_^a^	*<2.2* ^e^	*<1.3* ^e^	*<0.80* ^e^	*<2.8* ^e^	*<1.4* ^e^	*<0.81* ^e^	*<0.78* ^e^
Fe	C_t_^a^	*<2.2* ^e^	0.012 ± 0.001	0.014 ± 0.001	*<1.8* ^e^	*<1.5* ^e^	0.011 ± 0.002	0.012 ± 0.001
K	C_t_ ^a^	12.5 ± 0.2	12.0 ± 0.2	12.5 ± 0.1	11.6 ± 0.1	11.9 ± 0.1	11.1 ± 0.2	11.8 ± 0.1
	*F*_calculated_ ^b^		1.00	4.00	4.00	4.00	1.00	4.00
	∣*t*_calculated_∣ ^c^		3.062	0.000	6.971	4.648	8.573	5.422
Mg	C_t_ ^a^	14.7 ± 0.1	14.2 ± 0.2	14.8 ± 0.1	14.3 ± 0.2	14.7 ± 0.1	14.1 ± 0.3	14.3 ± 0.2
	*F*_calculated_ ^b^		4.00	1.00	4.00	1.00	9.00	4.00
	∣*t*_calculated_∣ ^c^		3.873	1.225	3.098	0.000	3.286	3.098
Mn	C_t_ ^a^	0.400 ± 0.003	0.374 ± 0.005	0.392 ± 0.002	0.365 ± 0.005	0.385 ± 0.004	0.367 ± 0.004	0.377 ± 0.002
	*F*_calculated_ ^b^		2.78	1.78	2.78	1.78	1.78	2.25
	∣*t*_calculated_∣ ^c^		7.723	2.771	10.397	5.196	11.432	11.049
Na	C_t_ ^a^	25.0 ± 0.2	24.8 ± 0.4	24.9 ± 0.2	22.9 ± 0.5	25.0 ± 0.4	23.1 ± 0.7	23.6 ± 0.2
	*F*_calculated_ ^b^		4.00	1.00	6.25	4.00	12.25	1.00
	∣*t*_calculated_∣ ^c^		0.775	0.612	6.754	0.000	4.520	8.573
Ni	C_t_^a^	*<11* ^e^	*<4.4* ^e^	*<3.1* ^e^	*<6.2* ^e^	*<4.6* ^e^	*<3.4* ^e^	*<3.4* ^e^
Pb	C_t_^a^	*<34* ^e^	*<10* ^e^	*<8.5* ^e^	*<16* ^e^	*<13* ^e^	*<11* ^e^	*<10* ^e^
Sr	C_t_ ^a^	0.480 ± 0.002	0.453 ± 0.004	0.474 ± 0.002	0.458 ± 0.005	0.460 ± 0.003	0.437 ± 0.003	0.438 ± 0.002
	*F*_calculated_ ^b^		1.78	1.00	2.78	1.00	1.00	1.00
	∣*t*_calculated_∣ ^c^		9.353	2.449	6.535	8.165	17.555	17.146
Zn	C_t_ ^a^	0.016 ± 0.001	0.014 ± 0.001	0.016 ± 0.001	0.012 ± 0.001	0.015 ± 0.001	0.010 ± 0.001	0.012 ± 0.001
	*F*_calculated_ ^b^		1.00	1.00	1.00	1.00	1.00	1.00
	∣*t*_calculated_∣ ^c^		2.449	0.000	4.899	1.225	7.348	4.899

P1: microwave-assisted closed-vessel digestion in concentrated HNO_3_. P2: acidification with concentrated HNO_3_ to 5%. P3: acidification with concentrated HNO_3_ to 5%, followed by the sonication (US) treatment. P4: 2-fold (1:1. *w/w*) dilution with 10% HNO_3_. P5: 2-fold (1:1. *w/w*) dilution with 10% HNO_3_, followed by the sonication (US) treatment. P6: direct analysis (no pretreatment). P7: direct analysis preceded by the sonication (US) treatment. ^a^ Total concentrations of elements (mean values (*n* = 3) ± standard deviations (SDs)) in µg g^−1^. ^b^ The critical value of the *F*-test (*F*_critical_): 19.00 (α = 0.05, *n* = 3, df = 2). ^c^ The critical value of the *t*-test (*t*_critical_*)*: 2.776 (α = 0.05, *n* = 3, df = 4). ^d^ The Cochran–Cox *C*-test with the critical value (*C_critical_*): 4.303 (α = 0.05, *n* = 3, df = 4) was used (*F*_calculated_ > *F*_critical_). ^e^ Below the method limit of detection (MLOD, ng g^−1^).

**Table 3:** Concentrations of elements and caffeine determined in the analyzed yerba mate (YM) energy drinks (YM1–YM11) by ICP-OES and UV spectrophotometry, respectively.

	Concentration ^a^, mg kg^−1^												
	YM1	YM2	YM3	BS4	YM5	YM6	YM7	YM8	YM9	YM10	YM11	Min.–Max.	Mean ^b^
Al	0.111 (0.93)	0.251 (0.39)	0.210 (0.95)	0.070 (2.9)	0.073 (1.4)	1.26 (0.95)	0.526 (0.76)	0.596 (0.36)	0.459 (0.21)	0.072 (2.7)	0.347 (1.3)	0.070–1.26	0.237
Ba	0.094 (1.2)	0.066 (1.7)	0.047 (2.2)	0.029 (3.5)	0.080 (1.2)	0.328 (1.8)	0.176 (0.66)	0.163 (0.73)	0.099 (1.2)	0.080 (1.4)	0.114 (1.0)	0.029–0.328	0.095
Ca	10.3 (1.2)	24.4 (1.2)	4.85 (2.1)	85.8 (2.7)	91.0 (0.44)	57.8 (1.3)	39.6 (0.76)	18.9 (2.6)	11.9 (0.84)	110 (0.91)	41.2 (0.97)	4.85–110	30.6
Cd	*<0.50* ^c^	*<0.50* ^c^	*<0.50* ^c^	*<0.50* ^c^	*<0.50* ^c^	*<0.50* ^c^	*<0.50* ^c^	*<0.50* ^c^	*<0.50* ^c^	*<0.50* ^c^	*<0.50* ^c^	---	---
Cr	*<0.80* ^c^	*<0.80* ^c^	*<0.80* ^c^	*<0.80* ^c^	*<0.80* ^c^	0.025 (4.0)	0.010 (10)	0.014 (7.1)	0.011 (9.1)	*<0.80* ^c^	0.011 (9.1)	0.010–0.025	0.013
Cu	*<0.80* ^c^	*<0.80* ^c^	0.028 (3.6)	*<0.80* ^c^	*<0.80* ^c^	0.026 (3.8)	0.089 (2.2)	0.042 (2.4)	0.013 (7.7)	*<0.80* ^c^	0.033 (3.0)	0.013–0.089	0.032
Fe	0.072 (2.8)	0.071 (3.3)	0.018 (5.6)	0.050 (3.1)	0.014 (7.1)	1.12 (0.63)	0.302 (0.21)	0.146 (1.9)	0.072 (1.9)	0.054 (3.7)	0.128 (1.1)	0.014–1.12	0.085
K	10.2 (2.9)	41.8 (0.96)	31.6 (2.8)	49.6 (2.4)	12.5 (0.80)	640 (1.4)	152 (2.0)	229 (4.4)	108 (0.93)	7.30 (0.82)	157 (0.64)	7.30–640	118
Mg	10.4 (1.9)	18.3 (1.1)	10.5 (1.9)	27.3 (3.7)	14.8 (0.68)	88.8 (1.1)	47.4 (0.84)	70.0 (4.3)	42.2 (0.94)	15.4 (1.3)	57.2 (0.70)	10.4–88.8	28.2
Mn	0.853 (2.5)	1.95 (0.54)	1.43 (0.76)	0.374 (1.4)	0.392 (0.51)	11.7 (1.1)	6.59 (1.0)	13.9 (0.86)	8.90 (0.89)	0.465 (2.3)	10.0 (0.56)	0.374–11.7	2.40
Na	12.0 (1.7)	5.40 (1.9)	8.90 (4.5)	101 (3.0)	24.9 (0.80)	6.20 (4.8)	5.30 (1.9)	84.1 (4.8)	5.30 (1.9)	22.6 (2.7)	36.1 (0.28)	5.30–101	16.0
Ni	0.060 (3.3)	0.080 (1.7)	*<3.1* ^c^	0.081 (3.7)	*<3.1* ^c^	0.134 (2.2)	0.091 (0.22)	0.090 (1.1)	0.050 (2.0)	0.058 (3.4)	0.099 (5.1)	0.050–0.134	0.076
Pb	*<8.5* ^c^	*<8.5* ^c^	*<8.5* ^c^	*<8.5* ^c^	*<8.5* ^c^	*<8.5* ^c^	*<8.5* ^c^	*<8.5* ^c^	*<8.5* ^c^	*<8.5* ^c^	*<8.5* ^c^	---	---
Sr	0.192 (1.6)	0.428 (0.47)	0.053 (1.9)	0.424 (0.26)	0.474 (0.42)	0.267 (0.44)	0.155 (0.65)	0.098 (1.0)	0.053 (1.9)	0.462 (0.22)	0.167 (0.60)	0.053–0.474	0.194
Zn	0.034 (6.5)	0.032 (4.0)	0.057 (2.3)	0.031 (4.2)	0.016 (6.2)	0.716 (1.1)	0.464 (1.0)	0.519 (0.21)	0.316 (0.70)	0.015 (10)	0.384 (0.60)	0.015–0.519	0.100
Caffeine ^d^	30.3 (0.33)	22.7 (0.44)	22.6 (2.2)	19.7 (1.3)	19.6 (2.5)	20.3 (2.3)	12.5 (0.80)	29.2 (1.4)	19.3 (1.0)	25.2 (0.40)	22.1 (0.90)	12.5–30.3	21.8
Caffeine ^e^	30	20	21	20	20	ND	20	ND	20	25	20	---	---

ND: not declared. ^a^ Average values (*n* = 3) with relative standard deviations (%RSDs) in brackets. ^b^ Geometric mean (mg kg^−1^). ^c^ Below the method detection limit (MLOD, ng g^−1^). ^d^ Average values (*n* = 3) in mg/100 mL with relative standard deviations (%RSDs) in brackets. ^e^ Caffeine content declared by the producer (in mg/100 mL).

**Table 4:** Concentrations ^a^ of elements and caffeine determined by ICP-OES and UV–VIS spectrophotometry, respectively, in the dialyzable and nondialyzable fractions of the analyzed yerba mate (YM) energy drinks (YM1–YM11).

	YM1	YM2	YM3	YM4	YM5	YM6	YM7	YM8	YM9	YM10	YM11
Al											
D: dialyzate ^b^	0.048 (4.2)	0.096 (3.2)	0.079 (5.1)	0.040 (2.5)	0.040 (2.5)	0.481 (1.2)	0.191 (1.6)	0.218 (5.0)	0.153 (5.2)	0.026 (3.8)	0.131 (1.5)
ND: nondialyzate ^c^	0.067 (1.5)	0.176 (0.57)	0.155 (0.65)	0.042 (2.4)	0.048 (4.2)	0.796 (1.3)	0.357 (0.28)	0.407 (0.74)	0.314 (0.64)	0.049 (3.1)	0.234 (4.2)
Agreement ^d^	104	108	111	117	121	101	104	105	102	104	105
Bioaccessibility ^e^	43.2	38.2	37.6	57.1	54.8	38.2	36.3	36.6	33.3	36.1	37.8
Ba											
D: dialyzate ^b^	0.049 (2.0)	0.03 (2.9)	0.025 (4.0)	0.018 (5.6)	0.049 (2.0)	0.202 (0.50)	0.099 (2.0)	0.092 (2.2)	0.051 (2.0)	0.048 (2.1)	0.066 (1.5)
ND: nondialyzate ^c^	0.041 (2.4)	0.029 (3.4)	0.019 (5.3)	0.011 (9.1)	0.040 (2.5)	0.148 (1.4)	0.072 (1.4)	0.076 (1.3)	0.044 (2.3)	0.034 (2.9)	0.050 (2.0)
Agreement ^d^	95.7	97.0	93.6	100	111	107	97.2	103	96.0	102	102
Bioaccessibility ^e^	52.1	53.0	53.2	62.1	61.3	61.6	56.3	56.4	51.5	60.0	57.9
Ca											
D: dialyzate ^b^	5.20 (1.5)	12.5 (4.0)	2.94 (0.34)	45.2 (0.22)	41.8 (0.48)	32.9 (4.3)	22.1 (0.45)	10.0 (4.7)	6.41 (1.7)	55.3 (2.0)	20.4 (0.49)
ND: nondialyzate ^c^	4.57 (3.3)	13.4 (0.75)	1.62 (1.9)	49.1 (0.20)	46.2 (0.43)	29.2 (2.7)	21.0 (2.9)	9.18 (4.0)	5.40 (1.7)	58.6 (0.51)	22.0 (3.6)
Agreement ^d^	94.9	106	94.0	110	96.7	107	109	101	99.2	104	103
Bioaccessibility ^e^	50.5	51.2	60.6	52.7	45.9	59.6	55.8	52.9	53.9	50.3	49.5
Fe											
D: dialyzate ^b^	0.035 (2.9)	0.028 (3.6)	0.006 (17)	0.015 (6.7)	0.005 (20)	0.576 (1.4)	0.109 (0.92)	0.051 (2.0)	0.030 (3.3)	0.026 (3.2)	0.057 (1.8)
ND: nondialyzate ^c^	0.035 (2.9)	0.046 (2.2)	0.013 (7.7)	0.034 (2.9)	0.011 (9.1)	0.601 (1.5)	0.211 (0.95)	0.088 (2.3)	0.041 (2.1)	0.027 (3.1)	0.076 (2.6)
Agreement ^d^	97.2	104	106	98.0	114	105	106	95.2	98.6	98.4	104
Bioaccessibility ^e^	48.8	39.5	33.3	30.4	32.9	51.4	36.1	34.9	42.4	48.4	44.5
Mg											
D: dialyzate ^b^	4.41 (0.68)	8.35 (3.1)	4.47 (4.5)	13.4 (1.5)	7.03 (1.6)	47.5 (2.5)	25.6 (0.39)	34.0 (3.2)	20.0 (3.0)	7.90 (0.76)	26.8 (1.5)
ND: nondialyzate ^c^	5.65 (0.68)	9.72 (0.31)	5.75 (1.4)	14.9 (0.67)	8.17 (0.49)	42.8 (2.1)	25.0 (0.40)	35.6 (1.1)	22.4 (0.45)	8.25 (0.73)	28.5 (0.70)
Agreement ^d^	97.1	98.9	97.1	104	103	102	107	99.4	100	105	96.7
Bioaccessibility ^e^	42.4	45.6	42.6	49.1	47.5	53.5	54.0	48.6	47.4	51.3	46.9
Mn											
D: dialyzate ^b^	0.338 (1.5)	0.837 (3.7)	0.594 (3.2)	0.178 (2.8)	0.185 (1.1)	5.80 (3.8)	3.04 (0.33)	6.60 (4.1)	3.89 (4.1)	0.223 (4.0)	4.62 (1.7)
ND: nondialyzate ^c^	0.500 (0,20)	1.12 (0.89)	0.847 (1.8)	0.218 (0.46)	0.234 (0.85)	6.45 (2.2)	3.47 (0.86)	7.92 (1.8)	5.00 (1.2)	0.255 (0.39)	5.62 (0.89)
Agreement ^d^	98.2	100	101	106	107	105	98.8	104	99.9	103	102
Bioaccessibility ^e^	39.6	42.9	41.5	47.6	47.2	49.6	46.1	47.5	43.7	48.0	46.2
Sr											
D: dialyzate ^b^	0.109 (0.92)	0.232 (3.9)	0.035 (2.9)	0.228 (2.6)	0.271 (2.6)	0.169 (3.6)	0.094 (1.1)	0.064 (3.1)	0.034 (2.9)	0.258 (2.7)	0.109 (1.8)
ND: nondialyzate ^c^	0.089 (2.2)	0.215 (0.47)	0.019 (5.3)	0.199 (1.0)	0.248 (0.80)	0.109 (2.8)	0.066 (4.5)	0.039 (5.1)	0.017 (5.9)	0.220 (1.8)	0.073 (2.7)
Agreement ^d^	103	104	102	101	110	104	103	105	96.2	104	92.4
Bioaccessibility ^e^	56.8	54.2	66.0	53.8	57.2	63.6	60.6	65.3	64.2	55.8	55.3
Zn											
D: dialyzate ^b^	0.017 (5.9)	0.019 (10)	0.031 (3.2)	0.017 (5.9)	0.009 (11)	0.401 (2.0)	0.241 (0.83)	0.282 (3.9)	0.154 (3.2)	0.007 (14)	0.191 (0.52)
ND: nondialyzate ^c^	0.020 (5.0)	0.018 (5.6)	0.029 (3.4)	0.019 (5.3)	0.008 (12)	0.357 (2.5)	0.253 (2.0)	0.248 (4.8)	0.172 (3.5)	0.010 (10)	0.210 (1.4)
Agreement ^d^	109	116	105	116	106	106	107	102	103	113	104
Bioaccessibility ^e^	50.0	59.4	54.4	54.8	50.8	56.0	51.9	54.3	48.7	42.4	49.7
Caffeine											
D: dialyzate ^b^	13.9 (0.72)	10.9 (0.92)	11.4 (1.8)	9.35 (0.43)	9.16 (6.2)	10.5 (1.9)	5.57 (1.4)	13.6 (0.74)	9.49 (0.84)	13.9 (1.4)	10.4 (1.9)
ND: nondialyzate ^c^	17.1 (0.58)	13.0 (0.77)	12.4 (0.81)	10.0 (1.0)	10.4 (5.7)	13.1 (0.76)	6.73 (0.15)	17.0 (0.59)	10.6 (1.9)	14.6 (0.68)	12.1 (0.83)
Agreement ^d^	102	105	106	97.9	99.8	102	98.5	101	104	113	102
Bioaccessibility ^e^	45.9	48.0	50.4	47.5	46.7	51.7	44.6	46.6	49.2	55.2	41.1

^a^ Average values (*n* = 3) with relative standard deviations (%RSDs) in brackets. ^b^ Concentrations of elements (in mg kg^−1^) or caffeine (in mg/100 mL) in the dialyzable fraction (D) after the gastrointestinal digestion procedure. ^c^ Concentrations of elements (in mg kg^−1^) or caffeine (in mg/100 mL) in the nondialyzable fraction (ND) after the gastrointestinal digestion procedure. ^d^ Calculated as [(D + ND)/C_t_] × 100 (in %), where C_t_ corresponds to the total concentrations of elements (in mg kg^−1^) or caffeine (in mg/100 mL) determined in the analyzed yerba mate (YM) energy drinks. ^e^ Contributions of the bioaccessible fraction (in %) of the studied elements or caffeine calculated as (D/C_t_) × 100.

## Data Availability

All the results are in the present work.

## Supplementary Materials

The following supporting information can be downloaded at: https://www.mdpi.com/article/10.3390/molecules28083374/s1. Table S1: Recoveries of elements obtained by ICP-OES in the solutions of YM5 prepared using reference (P1) and alternative (P2–P7) sample preparation procedures. Table S2: The nutritional value of the analyzed yerba mate (YM) energy drinks (YM1–YM11).

## References

[1] Heck C.I., De Mejia E.G. (2007). Yerba Mate Tea (*Ilex paraguariensis*): A Comprehensive Review on Chemistry, Health Implications, and Technological Considerations. J. Food Sci..

[2] Kujawska M. (2018). Yerba Mate (*Ilex paraguariensis*) Beverage: Nutraceutical Ingredient or Conveyor for the Intake of Medicinal Plants? Evidence from Paraguayan Folk Medicine. Evid. Based Complement. Altern. Med..

[3] Bastos Markowicz D.H., Fornari A.C., de Queiroz Y.S., Soares R., Torres E.A. (2005). The chlorogenic acid and caffeine content of yerba maté (*Ilex paraguariensis*) beverages. Acta Farm. Bonaer..

[4] Cordoba Bragança V.L., Melnikov P., Zanoni L.Z. (2011). Trace Elements in Different Brands of Yerba Mate Tea. Biol. Trace Elem. Res..

[5] Olivari I., Paz S., Gutiérrez A.J., González-Weller D., Hardisson A., Sagratini G., Rubio C. (2020). Macroelement, trace element, and toxic metal levels in leaves and infusions of yerba mate (*Ilex paraguariensis*). Environ. Sci. Pollut. Res..

[6] Tobaldini Frizon C.N., Perussello C.A., Alfredo J., Hoffmann-Ribani R. (2018). Novel Beverages of Yerba-Mate and Soy: Bioactive Compounds and Functional Properties. Beverages.

[7] Samoggia A., Landuzzi P., Vicién C.E. (2021). Market Expansion of Caffeine-Containing Products: Italian and Argentinian Yerba Mate Consumer Behavior and Health Perception. Int. J. Environ. Res. Public Health.

[8] Burris K.P., Harte F.M., Davidson P.M., Stewart C.N., Zivanovic S. (2012). Composition and bioactive properties of yerba mate (*Ilex paraguariensis* A. St.-Hill.: A review. Chil. J. Agric. Res..

[9] Oellig C., Schunck J., Schwack W. (2018). Determination of caffeine, theobromine and theophylline in Mate beer and Mate soft drinks by high-performance thin-layer chromatography. J. Chromatogr..

[10] Pozebon D., Dressler V.L., Marcelo M.C.A., de Oliveira T.C., Ferrão M.F. (2015). Toxic and nutrient elements in yerba mate (*Ilex paraguariensis*). Food Addit. Contam. Part B.

[11] Majewski Ulbrich N.C., do Prado L.L., Zimmer Barbosa J., Araujo E.M., Poggere G., Vargas Motta A.C., Prior S.A., Magri E., Young S.D., Broadley M.R. (2022). Multi-elemental Analysis and Health Risk Assessment of Commercial Yerba Mate from Brazil. Biol. Trace Elem. Res..

[12] Pereira C.C., Souza A.O., Oreste E.O., Cidade M.J.A., Cadore S., Ribeiro A.S., Vieira M.A. (2016). Acid Decomposition of Yerba Mate (*Ilex paraguariensis*) Using a Reflux System for the Evaluation of Al, Ca, Cd, Cr, Cu, Fe, K, Mg, Mn, Na, Pb and Zn Contents by Atomic Spectrometric Techniques. J. Braz. Chem. Soc..

[13] Janda K., Jakubczyk K., Łukomska A., Baranowska-Bosiacka I., Rębacz-Maron E., Dec K., Kochman J., Gutowska I. (2020). Effect of the Yerba mate (*Ilex paraguariensis*) brewing method on the content of selected elements and antioxidant potential of infusions. Pol. J. Chem. Technol..

[14] Szymczycha-Madeja A., Welna M., Pohl P. (2019). Method validation for multi-elemental analysis of dialyzable and non-dialyzable fraction of coffee brews by F AAS and ICP OES: A bioaccessibility study. Food Anal. Meth..

[15] Szymczycha-Madeja A., Welna M., Pohl P. (2020). Simplified method of multi-elemental analysis of dialyzable fraction of tea infusions by FAAS and ICP OES. Biol. Trace Elem. Res..

[16] Schmite B.F.P., Bitobrovec A., Hacke A.C.M., Pereira R.P., Weinert P.L., dos Anjos V.E. (2019). In vitro bioaccessibility of Al, Cu, Cd, and Pb following simulated gastrointestinal digestion and total content of these metals in different Brazilian brands of yerba mate tea. Food Chem..

[17] Marcelo M.C.A., Martins C.A., Pozebon D., Dressler V.L., Ferrão M.F. (2014). Classification of yerbamate (*Ilex paraguariensis*) according to the country of origin based on element concentrations. Microchem. J..

[18] Vargas Motta A.C., Zimmer Barbosa J., Magri E., Pedreira G.Q., Santin D., Prior S.A., Consalter R., Young S.D., Broadley M.R., Benedetti E.L. (2020). Elemental composition of yerba mate (*Ilex paraguariensis* A.St.-Hil.) under low input systems of southern Brazil. Sci. Total Environ..

[19] Alice Teresa Valduga A.T., Gonçalves I.L., Ederlan Magri E. (2019). Analysis of the Presence of Toxic Metals in Yerba Mate Samples: A Case Study from South Brazil. Water Air Soil Pollut..

[20] Zimmer Barbosa J., Zambon L.M., Vargas Motta A.C., Wendling I. (2015). Composition, hot-water solubility of elements and nutritional value of fruits and leaves of yerba mate. Ciênc. Agrotec. Lavras.

[21] Wróbel K., Wróbel K., Urbina E.M.C. (2000). Determination of Total Aluminum, Chromium, Copper, Iron, Manganese, and Nickel and Their Fractions Leached to the Infusions of Black Tea, Green Tea, *Hibiscus sabdariffa*, and *Ilex paraguariensis* (Mate) by ETA-AAS. Biol. Trace Elem. Res..

[22] Konieczka P., Namieśnik J. (2009). Quality Assurance and Quality Control in the Analytical Chemical Laboratory: A Practical Approach.

[23] Kolayli S., Ocak M., Kucuk M., Abbasoglu R. (2013). Does caffeine bind to metal ions?. Food Chem..

[24] Otten J.J., Hellwig J.P., Meyers L.D. (2006). Dietary Reference Intakes: The Essential Guide to Nutrient Requirements.

[25] WHO (1996). World Health Organization. Trace Elements in Human Nutrition and Health.

[26] SCHER—Scientific Committee on Health and Environmental Risks (2012). Assessment of the Tolerable Daily Intake of Barium.

[27] Pohl P., Bielawska-Pohl A., Dzimitrowicz A., Greda K., Jamroz P., Lesniewicz A., Szymczycha-Madeja A., Welna M. (2018). Understanding element composition of medicinal plants used in herbalism-a case study by analytical atomic spectrometry. J. Pharmaceut. Biomed..

