# Supplementary material for: Novel ICP-OES-Based Method for the Reliable Determination of the Total Content of 15 Elements in Yerba Mate Drinks along with the Determination of Caffeine and the In Vitro Bioaccessibility of the Compounds
Source: Molecules. 2023 Apr 11;28(8):3374. doi: 10.3390/molecules28083374 (PMC10145091; doi:10.3390/molecules28083374)
Supplement: Supplementary file 1 [file molecules-28-03374-s001.zip › molecules-2309984-supplementary.pdf]

Supplementary Materials

Table S1 Recoveries[a] of elements obtained by ICP OES in the solutions of the YM5 drink prepared using the reference (P1) and alternative (P2-P7) sample preparation procedures.

1.	Added, mg kg^{-1}	2. P1	3. P2	4. P3	5. P4	6. P5	7. P6	8. P7
	10. 0.050	11. 105 ± 2	12. 96.3 ± 3.2	13. 102 ± 1	14. 105 ± 9	15. 104 ± 9	16. 99.2 ± 0.6	17. 106 ± 1
9. Al	0.100	103 ± 1	98.2 ± 0.4	101 ± 1	98.9 ± 2.8	98.2 ± 2.8	99.2 ± 1.6	97.4 ± 0.8
	0.150	98.8 ± 2.5	99.6 ± 5.5	99.9 ± 0.3	100 ± 1	100 ± 1	100 ± 2	98.9 ± 1.1
	0.050	104 ± 1	99.2 ± 1.6	101 ± 1	99.6 ± 0.1	98.9 ± 0.1	99.5 ± 0.4	101 ± 1
Ba	0.100	104 ± 1	99.2 ± 0.6	100 ± 1	100 ± 2	99.6 ± 2.4	100 ± 1	92.1 ± 0.5
	0.150	99.0 ± 2.6	100 ± 1	99.8 ± 0.4	100 ± 1	100 ± 1	99.9 ± 0.3	97.0 ± 1.2
	0.100	98.0 ± 2.5	87.1 ± 1.9	99.8 ± 0.3	90.1 ± 2.4	90.9 ± 2.1	85.1 ± 1.4	112 ± 3
Ca[b]	0.250	103 ± 1	92.8 ± 3.0	102 ± 1	91.9 ± 1.2	96.6 ± 3.0	88.7 ± 0.8	94.0 ± 0.8
	0.500	102 ± 2	90.5 ± 1.2	100 ± 2	93.1 ± 3.8	94.3 ± 1.2	92.5 ± 1.9	95.5 ± 0.9
	0.050	105 ± 2.0	91.2 ± 7.3	105 ± 1	98.3 ± 0.4	95.2 ± 0.4	100 ± 2	88.6 ± 1.8
Cd	0.100	102 ± 1.0	96.3 ± 0.8	103 ± 1	99.9 ± 2.9	98.4 ± 2.8	100 ± 1	95.8 ± 0.6
	0.150	98.8 ± 2.3	97.9 ± 0.9	99.1 ± 0.6	99.8 ± 1.4	99.5 ± 1.5	99.9 ± 0.3	99.0 ± 1.2
	0.050	106 ± 2	78.0 ± 0.1	99.2 ± 0.9	96.2 ± 0.4	98.6 ± 0.4	99.2 ± 0.2	105 ± 1
Cr	0.100	101 ± 1	84.5 ± 0.9	100 ± 1	98.1 ± 2.7	99.0 ± 2.7	100 ± 1	96.4 ± 0.6
	0.150	98.6 ± 2.4	91.9 ± 0.9	100 ± 1	98.9 ± 2.2	99.6 ± 2.2	100 ± 1	99.1 ± 1.2
	0.050	105 ± 1	98.8 ± 3.3	102 ± 1	100 ± 1	99.3 ± 0.2	98.6 ± 1.7	105 ± 1
Cu	0.100	103 ± 1	99.9 ± 0.6	100 ± 1	100 ± 3	99.7 ± 3.0	100 ± 1	95.9 ± 0.6
	0.150	98.2 ± 2.1	100 ± 1	100 ± 1	100 ± 1	100 ± 2	100 ± 1	99.1 ± 0.6
	0.050	105 ± 3	97.6 ± 4.4	101 ± 1	96.6 ± 0.2	98.2 ± 0.2	95.3 ± 0.1	105 ± 1
Fe	0.100	102 ± 1	98.9 ± 0.5	100 ± 1	98.9 ± 3.2	99.3 ± 3.2	99.7 ± 1.4	93.7 ± 0.3
	0.150	98.7 ± 2.5	101 ± 2	100 ± 1	99.6 ± 1.2	99.3 ± 1.2	100 ± 1	99.2 ± 1.0
	0.100	95.6 ± 2.2	104 ± 2	99.9 ± 0.4	115 ± 2	102 ± 1	108 ± 3	104 ± 1
K[b]	0.250	103 ± 1	102 ± 1	101 ± 2	93.9 ± 3.1	101 ± 3	114 ± 2	98.3 ± 1.5
	0.500	100 ± 4	106 ± 2	103 ± 2	103 ± 2	97.8 ± 2.4	99.9 ± 0.9	102 ± 5
	0.100	103 ± 4	86.7 ± 2.2	101 ± 4	88.4 ± 1.3	98.8 ± 1.1	98.0 ± 1.7	103 ± 3
Mg[b]	0.250	99.4 ± 2.7	90.0 ± 2.0	100 ± 1	92.1 ±1.0	102± 2	97.7 ± 0.9	100 ± 1
	0.500	98.3 ± 0.9	91.9 ± 1.2	99.5 ± 0.3	90.2 ± 2.5	98.9 ± 0.5	99.5 ± 0.1	102 ± 2
	0.100	107 ± 2	98.6 ± 3.3	103 ± 1	98.5 ± 0.4	101 ± 1	101 ± 1	102 ± 1
Mn	0.250	99.1 ± 1.3	99.6 ± 1.4	101 ± 1	99.0 ± 1.3	99.1 ± 1.3	100 ± 1	98.2 ± 0.1
	0.500	98.6 ± 2.6	100 ± 1	99.3 ± 0.4	100 ± 1	100 ± 1	99.8 ± 0.2	99.6 ± 0.7
	0.100	103 ± 1	106 ± 1	100 ± 2	85.5 ± 1.2	97.9 ± 2.2	110 ± 2	109 ± 2
Na[b]	0.250	99.6 ± 1.7	103 ± 2	99.9 ± 1.5	91.9 ± 2.5	99.0± 1.9	103 ± 3	99.5± 1.0
	0.500	101 ± 1	104 ± 1	98.5 ± 0.7	93.3 ± 2.4	101 ± 5	114 ± 3	108 ± 1
	0.050	107 ± 4	76.8± 1.3	100 ± 2	98.9 ± 1.0	98.2 ± 1.0	101 ± 1	105 ± 1
Ni	0.100	107 ± 4	84.0± 0.6	100 ± 1	101 ± 2	100 ± 2	101 ± 1	97.5 ± 0.7
	0.150	98.6 ± 2.7	95.4± 1.0	99.9 ± 0.6	99.8 ± 1.7	100 ± 2	99.8 ± 0.2	99.0 ± 1.4
	0.050	105 ± 3	99.8 ± 2.9	98.3 ± 4.3	99.7 ± 2.2	97.7 ± 2.2	103 ± 5	104 ± 4
Pb	0.100	104 ± 2	96.0 ± 0.9	97.0 ± 3.4	101 ± 1	110 ±1	99.2 ± 2.0	94.9 ± 1.3
	0.150	98.9 ± 3.3	94.2 ± 0.6	99.2 ± 0.2	99.8 ± 3.0	98.6 ±2.9	100 ± 2	99.2 ± 0.3

Sr	0.100	105 ± 1	101 ± 2	101 ± 1	98.4 ± 0.4	99.0 ± 0.4	95.7 ± 0.1	103 ± 2
	0.250	103 ± 1	96.7 ± 0.7	100 ± 1	107 ± 2	100 ± 2	100 ± 1	97.8 ± 1.2
	0.500	99.8 ± 1.3	96.0 ± 0.5	100 ± 1	98.5 ± 0.9	100 ± 1	100 ± 1	99.4 ± 1.1
Zn	0.050	105 ± 5	100 ± 3	99.5 ± 1.0	100 ± 1	99.2 ± 1.2	99.9 ± 0.4	106 ± 1
	0.100	102 ± 1	99.2 ± 1.0	101 ± 1	99.8 ± 2.8	100 ± 3	100 ± 1	95.7 ± 0.6
	0.150	98.8 ± 2.4	98.8 ± 0.7	99.9 ± 0.6	100 ± 1	100 ± 1	100 ± 1	98.9 ± 1.5

P1: microwave-assisted closed-vessel digestion in concentrated HNO_3. P2: acidification with concentrated HNO_3 to 5%. P3: acidification with concentrated HNO_3 to 5% followed by the sonication (US) treatment. P4: 2-fold (1:1. w/w) dilution with 10% HNO_3. P5: 2-fold (1:1. w/w) dilution with 10% HNO_3 followed by the sonication (US) treatment. P6: direct analysis (no pre-treatment). P7: direct analysis preceded by the sonication (US) treatment.

[a] Average values (n=3) with standard deviations (SDs).

[b] Additions made to diluted (50- or 100-fold) samples of the YM5 drink.

Table S2. The nutritional value of the analyzed Yerba Mate (YM) energy drinks (YM1-YM11).

		RDI[a], %												
		YM1	YM2	YM3	BS4	YM5	YM6	YM7	YM8	YM9	YM10	YM11	Min.-max.	Mean[b]
Ca	M<br>F	0.52	1.2	0.29	4.5	4.2	3.8	2.4	1.1	0.71	6.1	2.2	0.29-4.2	1.7
Fe	M	0.44	0.35	0.07	0.19	0.06	7.9	1.5	0.70	0.42	0.36	0.78	0.06-7.9	0.44
	F	0.20	0.16	0.03	0.08	0.06	3.5	0.67	0.31	0.19	0.16	0.35	0.03-3.5	0.20
Mg	M	1.1	2.0	1.1	3.3	1.7	13	6.9	9.1	5.4	2.1	7.2	1.1-13	3.5
	F	1.4	2.6	1.4	4.3	2.2	17	8.9	12	7.0	2.8	9.4	1.4-17	4.5
Mn	M	15	36	26	7.7	8.0	278	145	316	186	11	221	7.7-316	50
	F	19	46	33	10	10	355	180	403	238	14	282	10-355	64
Zn	M	0.13	0.17	0.28	0.15	0.07	4.0	2.4	2.8	1.5	0.06	1.9	0.06-2.8	0.48
	F	0.18	0.24	0.39	0.21	0.10	5.5	3.3	3.9	2.1	0.09	2.6	0.09-3.9	0.67
Package, mL		500	500	500	500	500	330	330	330	330	330	330	---	---
Caffeine *per* serving[c], mg		70	54	57	47	46	35	18	45	31	46	34	18-70	42

M: male. F: female. RDI: recommended daily intake.[a] Coverage of the RDI for essential elements involved with the intake of 1 L of examined Yerba Mate (YM) drinks based on their concentration in the bioaccessible fraction and the respective dietary reference values, i.e. RDI (in mg *per* day) for healthy adults (male and female).

[b] Geometric mean (%).[c] Including the size of the analyzed Yerba Mate (YM) drinks and the caffeine content determined in the bioaccessible fraction.
